# The first survey of the beetles (Coleoptera) of the Farasan Archipelago of the southern Red Sea, Kingdom of Saudi Arabia

**DOI:** 10.3897/zookeys.959.51224

**Published:** 2020-08-14

**Authors:** Mahmoud S. Abdel-Dayem, Usama M. Abu El-Ghiet, Tarek M. Elsheikh, Ali A. Elgharbawy, Zarrag I.A. Al-Fifi, Hathal M. Aldhafer

**Affiliations:** 1 King Saud University Museum of Arthropods (KSMA), Plant Protection Department, College of Food and Agricultural Sciences, King Saud University, Riyadh, 11451, Saudi Arabia King Saud University Museum of Arthropods Riyadh Saudi Arabia; 2 Entomology Department, Faculty of Science, Cairo University, Giza, 12613, Egypt Cairo University Giza Egypt; 3 Biology Department, Faculty of Science, Jazan University, Jazan, Saudi Arabia Jazan University Jazan Saudi Arabia; 4 Plant Protection Department, Desert Research Center, Mataria, Cairo, Egypt Desert Research Center Cairo Egypt; 5 Zoology Department, Faculty of Science, Al-Azhar University, Cairo, Egypt Al-Azhar University Cairo Egypt

**Keywords:** Beetles, Farasan Islands, faunal survey, Saudi Arabia, zoogeography

## Abstract

The Farasan Archipelago is a group of small coral islands and islets in the southern Red Sea, offshore of the southwestern Kingdom of Saudi Arabia (KSA). These islands are internationally important as breeding sites for turtles and bird species and regionally for its threatened, rare, and endemic flora and other fauna. The beetles (Coleoptera) of the Archipelago have not been previously surveyed. This study presents the first data on the beetle fauna based on a recent survey of the Farasan Archipelago. In total, 179 beetle species (including three synanthropic species) in 145 genera and 31 coleopteran families were determined. The Carabidae are represented by 31 species, followed by the Tenebrionidae (22 species), Chrysomelidae (17 species), Scarabaeidae (13 species), and Coccinellidae (12 species). The genus *Lasiocera* Dejean, 1831 and the species *Amblystomus villiersanus* Bruneau de Miré, 1991 (Carabidae) are new for the beetle fauna of the Arabian Peninsula, and eighteen species are new country records for KSA. Sand dune habitats on the islands were inhabited by the greatest number of species in comparison with other habitats. Zoogeographically, the beetle fauna of the Archipelago was dominated by the representatives of the Saharo-Arabian and Afrotropical elements (74 spp., 41.0%). Fourteen species (7.8%) were recognized as cosmopolitan and subcosmopolitan. No species was known to be exclusively endemic to Farasan Archipelago. Eighteen species (10.1%) were endemic to Arabian Peninsula and KSA. Approximately 64.8% (116 spp.) of the archipelago beetle species is found on the KSA mainland and is most closely allied to the south and southwestern KSA regions (sharing 91 spp.). Comparisons of the beetle faunas of the Farasan and Socotra archipelagos indicate that 30 families, 70 genera, and 28 species are shared.

## Introduction

Geographically, the Red Sea is the world’s northernmost tropical sea, located between Africa and Asia. The Kingdom of Saudi Arabia (KSA) occupies ca. 80% of its eastern coastline, which includes ca. 1,150 islands (SGS 2017). The KSA islands in the southern part of the Red Sea occupy an interesting geographical and ecological position, due to location between East Africa and the Arabian Peninsula and being part of the northern desert belt (Hall et al. 2010; Mallon 2011). Additionally, the flora and fauna range from localized temperate endemics that occur as disjunctive extensions of the Horn of Africa Hotspot and the Eastern Afromontane Hotspot in the southwestern regions (Myers et al. 2000; Mallon 2011).

**Figure 1.**
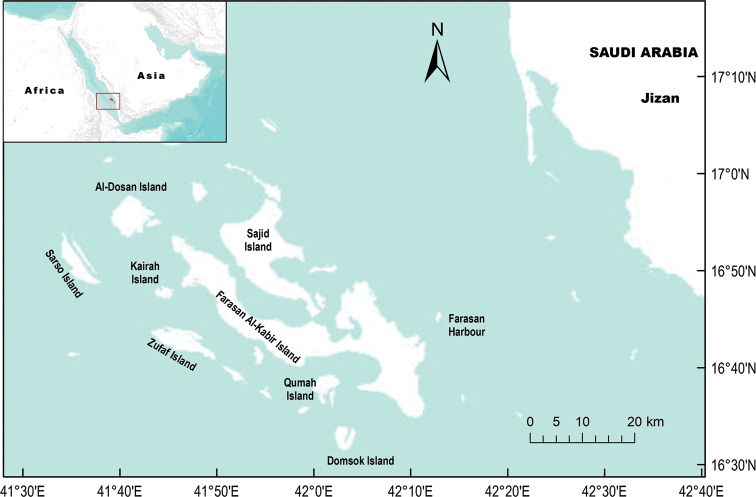
Study islands in Farasan Archipelago in the southern Red Sea, Kingdom of Saudi Arabia.

The Farasan Archipelago is located ca. 40 km offshore of the southwestern KSA coast near Jizan, between 16°20' to 17°20' latitude and 41°24' to 42°26' longitude (Fig. 1). The Archipelago is characterized by a fertile marine environment (Hausmann et al. 2019). It is an internationally important breeding site for turtles and several seabirds, including colonies of Crab Plover (*Dromas ardeola* Paykull, 1805), Greater Flamingo (*Phoenicopterus roseus* Pallas, 1811), Osprey (*Pandion haliaetus* (L., 1758)), Pink-Backed Pelican (*Pelecanus rufescens* Gmelin, 1789), Red-Billed Tropicbird (*Phaethon aethereus* Linnaeus, 1758), Sooty Falcon (*Falco concolor* Temminck, 1825), Saunders Little Tern (*Sternula albifrons* (Pallas, 1764)), White-Eyed Gull (*Ichthyaetus leucophthalmus* (Temminck, 1825)), and others (Thouless 1991; SWA 2019).

The Farasan Archipelago is recognized as having important floral communities, with numerous nationally and regionally rare, and endemic species (Kingdon 1990; Hall et al. 2010). However, its flora is not strikingly rich as compared to the Socotran Archipelago in the Indian Ocean, where 30% of the flora is endemic (Miller and Nyberg 1991). Most of the Farasan Archipelago has been declared as protected area since 1989 by the KSA government, mainly for conserving and restoring animal wildlife, especially the Idmi gazelle (*Gazella gazella farasani*) endemic to the islands and KSA, a species listed as vulnerable on the IUCN Red List (Cunningham and Wronski 2011).

In order to conserve the diversity of life, it is important to have a fundamental knowledge of what that biodiversity encompasses. The diversity and biogeography of flora have received considerably more attention than the fauna of the Farasan Archipelago. Currently available are several floristic studies conducted in the Archipelago (El-Demerdash 1996; Alfarhan et al. 2002; Atiqur Rahman et al. 2002; Hall et al. 2010; Tomas et al. 2010; Al Mutairi et al. 2012; Al Mutairi and Al-Shami 2014). There is some information on the vertebrates (birds: Jennings 1988; mammals: Habibi 1992; Masseti 2010; Cunningham and Wronski 2011; Wronski et al. 2012; and herpetofauna: Masseti 2014). The entomofauna knowledge of the Farasan Archipelago is relatively unknown with only published records for ants (Hymenoptera, Formicidae) (Sharaf and Al-Zailaie 2006), chewing lice (Phthiraptera, Menoponidae) (Alahmed et al. 2015), leucospid wasps (Hymenoptera, Leucospidae) (Gadallah et al. 2018), blow flies (Diptera, Calliphoridae) (Dawah et al. 2019), and for cuckoo wasps (Hymenoptera, Chrysididae) (Strumia and Dawah 2019). Additional entomological studies are needed to document insect diversity, composition, and especially for needed conservation assessments for these islands. Currently, no information is available on beetles of any KSA island in the Red Sea, contrary to nearby mainland of the Arabian Peninsula. Therefore, the objective of this study was to survey the beetles of the larger islands, Farasan Al-Kabir and Sajid, of the Farasan Archipelago and outline the zoogeographical features of this beetle fauna.

## Materials and methods

### Study area

The entire Farasan Archipelago (Fig. 1) consists of more than 120 coral reef islands of varying size (> 0.2–381 km^2^). The Archipelago reaches a width of ca. 120 km in south-east to north-west direction. The collective landmass of its islands covers ca. 710 km^2^ scattered across 5,408 km^2^ of sea (Cooper and Zazzaro 2014). Of the Archipelago’s islands, only two of them, Farasan Al-Kabir or Greater Farasan and Sajid or Saqid, are the two largest, and have permanent human residents (Almalki et al. 2017). Both islands together form the main landmass of the Archipelago and are connected together by a 300 m road bridge. The elevation ranges from 0 to 30 m with some hills rising up to 70 m. Farasan Al-Kabir is the largest of the islands, located in 16°42'N, 42°06'E, with a length of 66 km and a width of 6–8 km. It is 381 km^2^ in area and has the longest perimeter (216 km) (Fig. 1). Sajid Island to the northeast of Farasan Al-Kabir is the second largest island, with an area of approximately 150 km^2^ and a perimeter of 98 km. The islands are made up of elevated plateaus of Plio-Pleistocene coral reefs, limestone, and Aeolian deposits (Cooper and Zazzaro 2014). The characteristic features of the island soils are marine flats and dunes, and coral rock outcrops (Hall et al. 2010).

### Climate

**Figure 2.**
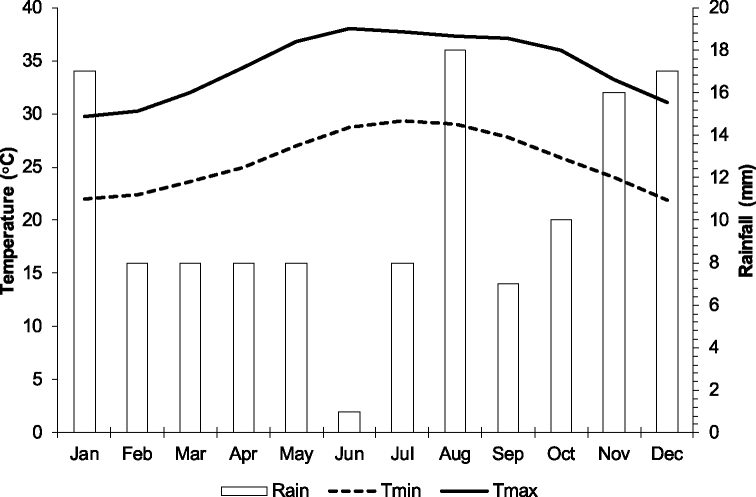
Climate data for Farasan Archipelago, southern Red Sea, Kingdom of Saudi Arabia (source of data http://www.worldclim.org).

There are no meteorological stations in any islands of the Farasan Archipelago, therefore climate data (temperature and rainfall) was obtained from the climate grids derived from WorldClim data set (http://www.worldclim.org). Arid and subtropical climate characterized the Archipelago, with a high annual average temperature (30.3 °C), a low rainfall and 64.2% average relative humidity. June to August is the hottest period of the year (28.7–38 °C) and December to February is the coolest (21.9–31.1 °C) (Fig. 2). The average annual precipitation of less than 50 mm, but being surrounded by the sea, the Archipelago is characterized with relatively high humidity present year around (NCWCD 2000). The most rain falls in January, August, November, and December (16–18 mm) (Fig. 2). The mean relative humidity in winter ranges from 70% to 80% and in summer between 65% and 78% (ALQthanin 2019).

### Flora

**Figure 3.**
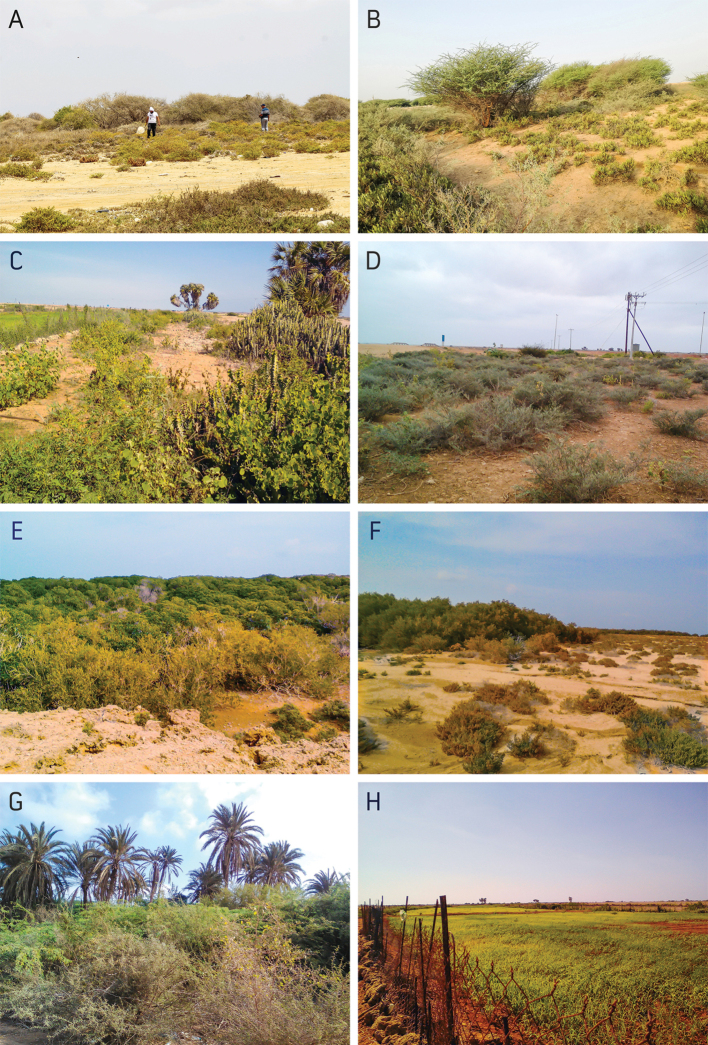
Photographs of characteristic plants of the Farasan Archipelago, Kingdom of Saudi Arabia **A***Acacia ehrenbergiana*, *Commiphora gileadensis*, *Limonium axillare*, and *Tetraena alba* in Al-Huseis **B***Acacia ehrenbergiana*, *Indigofera oblongifolia* and *Zygophyllum coccineum* in Khotp **C***Hyphaene thebaica*, *Caralluma acutangula*, *Euphorbia fractiflexa*, *Senna alexandrina*, and *Capparis sinaica* in Khotp **D***Indigofera oblongifolia* at Bridge **E***Rhizophora mucronata***F***Avicennia marina* in Al-Huseis **G***Acacia ehrenbergiana* and *Phoenix dactylifera* in sand Sajid **H** sorghum field.

The vascular plant flora of Farasan Archipelago includes around 200 species belonging to 129 genera and 53 families (Alfarhan et al. 2002; Al Mutairi and Al-Shami 2014). Farasan Al-Kabir Island harbours more than 70% of the Archipelago flora (Al Mutairi and Al-Shami 2014). The overall vegetation of the Archipelago is similar to that of the Tihama Plain of the southwestern KSA. The most prominent feature of the flora is the mangrove stands *Avicennia marina* (Forssk.) Vierh. that grow along the inlets and bays of Farasan Al-Kabir and Sajid islands (Fig. 3F) and intermixed with other mangrove species, *Rhizophora mucronata* Lam. (Fig. 3E). The halophytic communities in the sandy beaches dominated by *Aeluropus lagopoides* (L.) Trin., *Halopeplis perfoliata* (Forssk.) Bunge ex Asch., *Limonium axillare* (Forssk.) O. Kuntze (Fig. 3A), *Suaeda monoica* Forssk., *Tetraena alba* (L.f.) Beier & Thulin (Fig. 3A) and *Zygophyllum coccineum* L. (Fig. 3B). The vegetation in the interior regions includes small trees and shrubs of such as *Acacia ehrenbergiana* Hayne (Fig. 3A, G), *Aerva javanica* (Burm.f.) Juss. ex Schultes., *Blepharis edulis* Forssk., *Cadaba glandulosa* Forssk., *Capparis sinaica* Veill. (Fig. 3C), *Commiphora gileadensis* C. Christ. (Fig. 3A), *Grewia tenax* (Forssk.) Fiori., *Hyphaene thebaica* (L.) Mart (Fig. 3C), *Indigofera oblongifolia* Forssk. (Fig. 3B, D), *Ochradenus baccatus* Del., *Salvadora persica* L., and *Senna alexandrina* Miller (Fig. 3C); climbers such as *Cissus quadrangularis* L. and *Pentatropis nivalis* (J.F. Gmel.) D.V. Field & JRI Wood; and annuals such as *Tetraena simplex* (L.) Beier & Thulin, *Polycarpaea spicata* Wight, *Euphorbia granulata* Forssk. Throughout the area, grasses such as *Dichanthium foveolatum* (Del.) Roberty, *Hyparrhenia hirta* (L.) Stapf., *Panicum turgidum* Forssk., *Sporobolus helvolus* (Trin.) Dur. & Schinz. and *Tricholaena teneriffae* (L.f.) Link. are present. Additionally, there are two succulent species, *Caralluma acutangula* (Decne.) and *Euphorbia fractiflexa* S. Carter & JRI Wood (Fig. 3C), occur in the limestone areas.

Agricultural activities are limited and include cultivation of date palms (Fig. 3G), sorghum (Fig. 3H), watermelon, with a few herds of camels and goats. Recently, there has been a decrease in agricultural actives, and abandoned former agricultural fields are common sights on Sajid Island.

### Beetle collections

The beetle survey was conducted within the two largest islands, Farasan Al-Kabir and Sajid of Farasan Archipelago. Adult beetles were collected between January and December of 2017 using pitfall traps (PT), light traps (LT), and sweeping nets (SW). A voucher collection of all beetle species was deposited in the King Saud Museum of Arthropods (KSMA), College of Food and Agricultural Sciences, King Saud University, KSA. Beetles that we or other experts could not reliably determine to species were listed at genus level but were included in the study if specimens were considered morphologically different from related species.

### Data arrangement

The suborder, superfamily and family level classification and arrangement used here is that of Löbl and Smetana (2003–2008). The genera and species are arranged alphabetically under each family. Data on the general distribution is based mainly on Catalogue of Palaearctic Coleoptera (Löbl and Smetana 2003–2008) and Global Biodiversity Information Facility (GBIF: http://www.gbif.org). The distribution of each species in various zoogeographical regions is also indicated as a letter code (see “Abbreviations”) corresponding to main zoogeographic regions of the world proposed by Holt et al. (2013). The province distribution records of species shown with literature records are based on the published data in the series “Fauna of Saudi Arabia”, being published as part of the “Fauna of Arabia” (1979 to date), and other sources on KSA beetles.

### Abbreviations of zoogeographical distributions

**AFR** Afrotropical;

**AUS** Australian;

**COS** Cosmopolitan;

**MAD** Madagascan;

**ORR** Oriental;

**PAL** Palaearctic;

**SAR** Saharo-Arabian;

**SCO** Subcosmopolitan;

**SJP** Sino-Japanese.

## Results

### Checklist

#### Suborder Adephaga

**Superfamily Caraboidea**

**Family Carabidae**

**1. *Abacetus crenulatus* Dejean, 1831**

**Material examined. Farasan Island**: Al-Huseis, 16°45'20.8"N, 42°03'59.6"E, 7 m, 17.ii.2017, 1 (PT). Farasan Reserve, 16°42'26.3"N, 42°03'59.4"E, 5 m, 17.ii.2017, 1 (PT); 11.viii.2017, 1 (PT). Farasan Reserve, 16°42'26.5"N, 42°04'19.2"E, 7 m, 5.iii.2017, 2 (PT); 11.viii.2017, 1 (PT). **Sajid Island**: Bridge, 16°51'27.4"N, 41°55'49.0"E, 2 m, 10.vi.2017, 1 (PT); 28.vii.2017, 2 (PT). Khotp, 16°52'27.9"N, 41°54'43.9"E, 9 m, 25.i.2017, 1 (PT); 17.ii.2017, 2 (PT); 5.iii.2017, 9 (PT); 28.vii.2017, 4 (PT); 10.xi.2017, 1 (PT). Khotp, 16°52'47.6"N, 41°54'39.7"E, 11 m, 17.ii.2017, 2 (PT); 28.vii.2017, 14 (PT); 13.x.2017, 25 (PT). Sajid, 16°45'42.6"N, 41°59'56.0"E, 6 m, 17.ii.2017, 2 (PT); 10.vi.2017, 1 (PT); 28.vii.2017, 13 (PT); 2.xii.2017, 2 (PT).

**Literature records.** Asir and Baha (Abdel-Dayem et al. 2019).

**General distribution.**AFR species distributed in western and central of Sub-Saharan Africa, eastwardly to Arabian Peninsula (KSA).

**Remarks.** This species was collected by PT under the canopies of *Lim. axillare* in coastal sandy areas, *Ind. oblongifolia* in limestone areas and sand dunes, *Ble. edulis* and *Tet. alba* in salt marshes. Identification by M.S. Abdel-Dayem.

**2. *Amblystomus villiersanus* Bruneau de Miré, 1991**

**Material examined. Sajid Island**: Sajid, 16°45'42.6"N, 41°59'56.0"E, 6 m, 13.x.2017, 8 (LT); 10.xi.2017, 9 (LT); 2.xii.2017, 1 (LT).

**General distribution.**AFR species known from central Sahel (Chad, Niger) and Horn of Africa (Somalia). First record for Arabian Peninsula (KSA).

**Remarks.** The species was often frequently collected by LT set in planted palm tree orchards, sorghum, and *Aca. ehrenbergiana* in sand dunes. Identification by S. Facchini

**3. *Amblystomus* sp.**

**Material examined. Sajid Island**: Khotp, 16°52'27.9"N, 41°54'43.9"E, 9 m, 25.i.2017, 12 (SW); 2.xii.2017, 1 (SW). Sajid, 16°45'42.6"N, 41°59'56.0"E, 6 m, 25.i.2017, 35 (LT); 17.ii.2017, 10 (LT); 5.iii.2017, 6 (LT); 10.vi.2017, 4 (LT); 13.x.2017, 3 (LT); 2.xii.2017, 43 (LT).

**Remarks.** The specimens were collected by LT in sand dunes and by SW on *Ind. oblongifolia* in limestone areas.

**4. *Brachinus exhalans* (P. Rossi, 1792)**

**Material examined. Sajid Island**: Bridge, 16°51'27.4"N, 41°55'49.0"E, 2 m, 25.i.2017, 1 (PT). Khotp, 16°52'27.9"N, 41°54'43.9"E, 9 m, 25.i.2017, 1 (PT); 18.ii.2017, 4 (PT); 5.iii.2017, 72 (PT); 5.ix.2017, 1 (PT); 10.xi.2017, 4 (PT). Khotp, 16°52'47.6"N, 41°54'39.7"E, 11 m, 18.ii.2017, 2 (PT); 28.iv.2017, 2 (PT). Sajid, 16°45'42.6"N, 41°59'56.0"E, 6 m, 18.ii.2017, 1 (PT); 13.x.2017, 1 (PT).

**Literature records.** Makkah (Britton 1948).

**General distribution.** PAL_SAR species, distributed in Mediterranean Basin, east to Central Asia, Arabian Peninsula (KSA), Iraq and Iran.

**Remarks.** The species was frequently collected by PT in limestone areas and sand dunes. Identification by M.S. Abdel-Dayem.

**5. *Brachinus nobilis* Dejean, 1831**

**Material examined. Sajid Island**: Sajid, 16°45'42.6"N, 41°59'56.0"E, 6 m, 2.xii.2017, 1 (LT).

**Literature records.** Makkah and Riyadh (Britton 1948; Basilewsky 1979).

**General distribution.** AFR_SAR species, distributed in south Africa (Namibia, South Africa), Atlas Mountains in North Africa (Morocco, Algeria, Tunisia), east in Levant, Arabian Peninsula (KSA, Yemen (Socotra)), and Iran.

**Remarks.** The listed specimen was collected by LT in a sand dune. Identification by M.S. Abdel-Dayem.

**6. *Cephalota littorea* (Forskål, 1775)**

**Material examined. Sajid Island**: Sajid, 16°45'42.6"N, 41°59'56.0"E, 6 m, 10.xi.2017, 2 (LT).

**Literature records.** Asir and Jizan (Britton 1948).

**General distribution.**SAR species, distributed in Levant and Arabian Peninsula (KSA).

**Remarks.** The unique specimen was collected by LT in a sand dune. Identification by M.S. Abdel-Dayem.

**7. *Chlaenius* sp.**

**Material examined. Sajid Island**: Sajid, 16°45'42.6"N, 41°59'56.0"E, 6 m, 2.xii.2017, 1 (LT).

**Remarks.** The single listed specimen was collected by LT from a sand dune.

**8. *Chlaenius* sp.**

**Material examined. Sajid Island**: Khotp, 16°52'27.9"N, 41°54'43.9"E, 9 m, 25.i.2017, 1 (PT); 18.ii.2017, 3 (PT); 5.iii.2017, 9 (PT). Khotp, 16°52'47.6"N, 41°54'39.7"E, 11 m, 25.i.2017, 1 (PT); 18.ii.2017, 2 (PT).

**Remarks.** The specimens were collected by PT under the canopies of *Ind. oblongifolia* and *Cap. sinaica* in limestone areas.

**9. *Crasodactylus punctatus* Guérin-Méneville, 1847**

**Material examined. Sajid Island**: Sajid, 16°45'42.6"N, 41°59'56.0"E, 6 m, 28.vii.2017, 1 (LT).

**Literature records.** Asir and Baha (Abdel-Dayem et al. 2019).

**General distribution.** AFR_SAR species, distributed in Sahel Africa, northwards to North Africa (Algeria, Tunisia), east from Arabian Peninsula (KSA, Oman, Yemen (Socotra)), Iraq, and Iran to Pakistan.

**Remarks.** The single specimen was collected by LT from a sand dune. Identification by D.W. Wrase.

**10. *Cymbionotum semelederi* (Chaudoir, 1861)**

**Material examined. Sajid Island**: Khotp, 16°52'27.9"N, 41°54'43.9"E, 9 m, 12.viii.2017, 1 (PT). Khotp, 16°52'47.6"N, 41°54'39.7"E, 11 m, 17.ii.2017, 1 (PT); 12.viii.2017, 2 (PT). Sajid, 16°45'42.6"N, 41°59'56.0"E, 6 m, 17.ii.2017, 2 (PT); 5.iii.2017, 1 (LT).

**Literature records.** Eastern Province and Makkah (Britton 1948; Basilewsky 1979).

**General distribution.** AFR_SAR species, distributed throughout whole North Africa, east from Levant, Arabian Peninsula (KSA, Yemen) and Iran to Pakistan, northwards in Central Asia and south Caucasus, southwards to Sahel and Horn of Africa (Somalia).

**Remarks.** This species was collected by PT under the canopies of *Ind. oblongifolia* in limestone areas and *Sen. alexandrina* from sand dunes, and by LT in sand dunes. Identification by A.A. Elgharbawy.

**11. *Distichus planus* (Bonelli, 1813)**

**Material examined. Farasan Island**: Farasan Reserve, 16°42'26.3"N, 42°03'59.4"E, 5 m, 17.ii.2017, 1 (PT). Farasan Reserve, 16°42'26.5"N, 42°04'19.2"E, 7 m, 17.ii.2017, 1 (PT). **Sajid Island**: Bridge, 16°51'27.4"N, 41°55'49.0"E, 2 m, 17.ii.2017, 2 (PT). Khotp, 16°52'27.9"N, 41°54'43.9"E, 9 m, 17.ii.2017, 2 (PT); 5.iii.2017, 2 (PT); 10.vi.2017, 1 (PT). Khotp, 16°52'47.6"N, 41°54'39.7"E, 11 m, 17.ii.2017, 2 (PT); 28.vii.2017, 1 (PT). Sajid, 16°45'42.6"N, 41°59'56.0"E, 6 m, 17.ii.2017, 1 (PT).

**Literature records.** Riyadh (Abdel-Dayem et al. 2017).

**General distribution.** PAL_SAR species, distributed in the southern Europe, Central Asia, southwards to North Africa, Levant, Arabian Peninsula (KSA, Yemen), Iran to Pakistan.

**Remarks.** This species was collected by PT under the canopies of *Sud. monoica* and *Tet. alba* in salt marshes, *Ind. oblongifolia* in limestone areas and sand dunes; and *Sen. alexandrina* from sand dunes. Identification by M. Balkenohl.

**12. *Glycia rufolimbata* Maindron, 1905**

**Material examined. Sajid Island**: Sajid, 16°45'42.6"N, 41°59'56.0"E, 6 m, 5.ix.2017, 1 (LT).

**Literature records.** Asir, Jizan, Makkah (Britton 1948; Mateu 1986).

**General distribution.** AFR_SAR species, occurring in Sahel (Mauritania, Niger) east to Sudan and Horn of Africa, Arabian Peninsula (KSA), east to Iran and Afghanistan; northwards to Egypt, Levant, and reaching to Greece.

**Remarks.** The single specimen was collected by LT from a sand dune. Identification by R. Flex.

**13. *Glycia spencei* (Gistel, 1838)**

**Material examined. Farasan Island**: Al-Kosar, 16°40'14.8"N, 42°08'54.9"E, 7 m, 17.ii.2017, 1 (LT). Farasan Reserve, 16°42'26.3"N, 42°03'59.4"E, 5 m, 17.ii.2017, 1 (LT). **Sajid Island**: Sajid, 16°45'42.6"N, 41°59'56.0"E, 6 m, 25.i.2017, 1 (SW); 10.xi.2017, 8 (LT).

**Literature records.**Britton (1948) reported this species as *Glycia ornata* Klug, 1832 from Makkah.

**General distribution.** AFR_PAL_SAR species, distributed in the southern parts of the Palaearctic region, from southern Europe to south Caucasus and central Asia; North Africa, Levant, Arabian Peninsula (KSA, Yemen), and Iran to Pakistan; Horn of Africa and Sudan.

**Remarks.** The species was collected by LT in salt marshes and sand dunes and by SW on flowering *Sen. alexandrina* in sand dunes. Identification by R. Flex.

**14. *Lasiocera* sp.**

**Material examined. Sajid Island**: Sajid, 16°45'42.6"N, 41°59'56.0"E, 6 m, 10.xi.2017, 1 (LT).

**Remarks.** The genus *Lasiocera* Dejean, 1831 includes nearly 14 species, 12 of them distributed in Sub-Saharan Africa and two species in Southeast Asia (India, Pakistan, Seri Lanka) (Anichtchenko et al. 2007–2018). This record represents the first occurrence of the genus from Arabian Peninsula (KSA). It was collected by LT from sand dunes.

**15. *Lipostratia distinguenda* (Fairmaire, 1886)**

**Material examined. Sajid Island**: Khotp, 16°52'27.9"N, 41°54'43.9"E, 9 m, 25.i.2017, 4 (SW). Khotp, 16°52'47.6"N, 41°54'39.7"E, 11 m, 27.i.2017, 1 (SW). Sajid, 16°45'42.6"N, 41°59'56.0"E, 6 m, 10.xi.2017, 5 (LT).

**Literature records.** Jizan and Makkah (Britton 1948; Mateu 1986).

**General distribution.**AFR species, occurring in Horn of Africa and southern of Arabian Peninsula (KSA, Yemen (Socotra)).

**Remarks.** The specimens were collected by LT in sand dunes and by SW on *Ind. oblongifolia* in limestone areas. Identification by I. Rassol and R. Flex.

**16. *Mesolestes* sp.**

**Material examined. Sajid Island**: Bridge, 16°51'27.4"N, 41°55'49.0"E, 2 m, 25.i.2017, 2 (SW). Sajid, 16°45'42.6"N, 41°59'56.0"E, 6 m, 25.i.2017, 2 (LT).

**Remarks.** This species was collected by SW on *Abutilon pannosum*, *Aer. javanica*, *Ind. oblongifolia*, and by LT in sand dunes.

**17. Metadromius cf. brittoni (Basilewsky, 1948)**

**Material examined. Sajid Island**: Sajid, 16°45'42.6"N, 41°59'56.0"E, 6 m, 13.x.2017, 1 (LT).

**Literature records.** Asir, Baha, Jizan, and Riyadh (Abdel-Dayem et al. 2019).

**General distribution.**SAR species, distributed in Arabian Peninsula (KSA, Yemen) and Jordan.

**Remarks.** The single specimen was collected by LT from a sand dune. Identification by I. Rasool.

**18. *Microcosmodes arabicus* Häckel & Azadbakhsh, 2016**

**Material examined. Sajid Island**: Khotp, 16°52'27.9"N, 41°54'43.9"E, 9 m, 10.vi.2017, 1 (SW); 5.ix.2017, 1 (SW).

**Literature records.** Baha (Abdel-Dayem et al. 2019).

**General distribution.** END_AR species, known only from the southern Arabian Peninsula (KSA, Oman, Yemen).

**Remarks.** The species was collected by SW on *Cap. sinaica*, *Ind. oblongifolia*, and *I. spinosa* in limestone areas. Identification by M. Häackel.

**19. *Microlestes* sp.**

**Material examined. Sajid Island**: Khotp, 16°52'47.6"N, 41°54'39.7"E, 11 m, 25.i.2017, 2 (SW). Sajid, 16°45'42.6"N, 41°59'56.0"E, 6 m, 25.i.2017, 4 (LT); 18.ii.2017, 6 (LT); 5.iii.2017, 2 (LT); 28.iv.2017, 4 (LT); 23.v.2017, 4 (LT); 5.ix.2017, 4 (LT); 13.x.2017, 2 (LT); 10.xi.2017, 3 (LT).

**Remarks.** This species was collected by LT in limestone areas and sand dunes, and by SW on *Abu. pannosum* and *Aer. javanica*.

**20. *Myriochile melancholica* (Fabricius, 1798)**

**Material examined. Sajid Island**: Khotp, 16°52'27.9"N, 41°54'43.9"E, 9 m, 5.iii.2017, 1 (PT). Khotp, 16°52'47.6"N, 41°54'39.7"E, 11 m, 18.ii.2017, 1 (PT). Sajid, 16°45'42.6"N, 41°59'56.0"E, 6 m, 13.x.2017, 1 (LT); 10.xi.2017, 2 (LT).

**Literature records.** Asir, Baha, Jizan Najran, and Riyadh (Abdel-Dayem et al. 2019).

**General distribution.** AFR_MAD_ORR_PAL_SAR species, widely distributed in tropical Africa; North Africa to Levant and the entire Arabian Peninsula, from Iran to India and China; southern Europe to central Asia; and extending northwards to western Europe (France).

**Remarks.** This species was collected by LT in sand dunes, and by PT under the canopies on *Ind. oblongifolia* in limestone areas. Identification by M.S. Abdel-Dayem.

**21. *Poecilus wollastoni* (Wollaston, 1854)**

**Material examined. Sajid Island**: Khotp, 16°52'27.9"N, 41°54'43.9"E, 9 m, 5.iii.2017, 2 (PT); 2.xii.2017, 2 (PT). Sajid, 16°45'42.6"N, 41°59'56.0"E, 6 m, 23.v.2017, 2 (LT); 23.v.2017, 2 (PT); 2.xii.2017, 2 (LT).

**Literature records.** Riyadh (Abdel-Dayem et al. 2017).

**General distribution.**SAR species, distributed through North Africa, Canary Islands, and Morocco eastwardly to Egypt, Levant, Arabian Peninsula (KSA, Kuwait, Yemen), Iran, and Pakistan.

**Remarks.** The listed specimens of this species were collected by LT in sand dunes and by PT under *Ind. oblongifolia* in limestone areas and under *Sen. alexandrina* in sand dunes. Identification by M.S. Abdel-Dayem.

**22. *Pogonus gilvipes* Dejean, 1828**

**Material examined. Sajid Island**: Khotp, 16°52'27.9"N, 41°54'43.9"E, 9 m, 2.xii.2017, 1 (SW). Sajid, 16°45'42.6"N, 41°59'56.0"E, 6 m, 17.ii.2017, 3 (LT); 13.x.2017, 8 (LT); 10.xi.2017, 21 (LT); 2.xii.2017, 236 (LT).

**Literature records.** Makkah (Britton 1948).

**General distribution.** PAL_SAR species, distributed throughout North Africa, from Canary Islands east to Egypt, Levant, and Arabian Peninsula (KSA, Yemen), northward to southern, western, and eastern Europe, south European territory of Russia and central Asia (Uzbekistan).

**Remarks.** The species was collected by LT in sand dunes and by SW on *Sen. alexandrina* in limestone areas. Identification by M.S. Abdel-Dayem.

**23. *Siagona europaea* Dejean, 1826**

**Material examined. Sajid Island**: Al-Kosar, 16°40'14.8"N, 42°08'54.9"E, 7 m, 5.iii.2017, 2 (PT). Bridge, 16°51'27.4"N, 41°55'49.0"E, 2 m, 18.ii.2017, 1 (PT). Khotp, 16°52'27.9"N, 41°54'43.9"E, 9 m, 18.ii.2017, 1 (PT); 5.iii.2017, 2 (PT). Khotp, 16°52'47.6"N, 41°54'39.7"E, 11 m, 18.ii.2017, 1 (PT); 10.iv.2017, 1 (PT). Sajid, 16°45'42.6"N, 41°59'56.0"E, 6 m, 18.ii.2017, 2 (PT); 5.iii.2017, 2 (PT).

**Literature records.** Makkah and Riyadh (Abdel-Dayem et al. 2017).

**General distribution.** ORR_PAL_SAR species, spreading through southern Europe to Balkans and central Asia, southward from Levant, Arabian Peninsula (KSA, Yemen) and Iran to India.

**Remarks.** The specimens were collected by PT under the canopies of *Ind. oblongifolia* in limestone areas, *Sen. alexandrina* in sand dunes. Identification by A.A. Elgharbawy.

**24. *Sirdenus grayii* (Wollaston, 1862)**

**Material examined. Sajid Island**: Sajid, 16°45'42.6"N, 41°59'56.0"E, 6 m, 10.xi.2017, 1 (LT).

**General distribution.** AFR_ORR_PAL_SAR species, distributed from Canary Islands through Sahara Desert; east to Egypt, Levant, Arabian Peninsula (United Arab Emirates), Iran and India; northward to south Europe (Italy, Spain) and central Asia (Turkmenistan); southward to Africa (Cabo Verde, Gambia, Senegal). New country record for KSA.

**Remarks.** This unique specimen was collected by LT from a sand dune. Identification by M.S. Abdel-Dayem.

**25. *Stenolophus* sp.**

**Material examined. Sajid Island**: Sajid, 16°45'42.6"N, 41°59'56.0"E, 6 m, 25.i.2017, 18 (SW); 17.ii.2017, 1 (LT); 5.iii.2017, 3 (LT); 10.vi.2017, 7 (LT); 28.vii.2017, 1 (LT); 13.x.2017, 14 (LT); 14.x.2017, 1 (PT); 10.xi.2017, 7 (LT); 2.xii.2017, 1 (LT).

**Remarks.** The listed specimens were frequently collected from sand dunes by SW on *Sen. alexandrina* and by LT in old palm tree field.

**26. *Tachys gilvus* Schaum, 1863**

**Material examined. Sajid Island**: Sajid, 16°45'42.6"N, 41°59'56.0"E, 6 m, 10.xi.2017, 1 (LT).

**General distribution.** AFR_MAD species, widely distributed in tropical Africa, Madagascar, and cited from Egypt and Yemen. New record for KSA.

**Remarks.** This unique specimen was collected by LT in a sand dune. Identification by M.S. Abdel-Dayem.

**27. *Tachys* sp.**

**Material examined. Sajid Island**: Sajid, 16°45'42.6"N, 41°59'56.0"E, 6 m, 25.i.2017, 2 (LT); 17.ii.2017, 10 (LT); 5.iii.2017, 5 (LT); 28.iv.2017, 1 (LT); 13.x.2017, 10 (LT); 10.xi.2017, 1 (LT).

**Remarks.** This species was frequently collected by LT in sand dunes.

**28. *Tachyura* sp.**

**Material examined. Sajid Island**: Sajid, 16°45'42.6"N, 41°59'56.0"E, 6 m, 25.i.2017, 6 (LT); 17.ii.2017, 3 (LT); 5.iii.2017, 3 (LT); 28.iv.2017, 2 (LT); 23.v.2017, 1 (LT); 5.ix.2017, 5 (LT); 13.x.2017, 3 (LT); 10.xi.2017, 4 (LT).

**Remarks.** The listed specimens were frequently collected by LT in sand dunes.

**29. *Tachyura* sp.**

**Material examined. Sajid Island**: Sajid, 16°45'42.6"N, 41°59'56.0"E, 6 m, 25.i.2017, 6 (LT); 17.ii.2017, 3 (LT); 5.iii.2017, 4 (LT); 28.iv.2017, 3 (LT); 23.v.2017, 1 (LT); 5.ix.2017, 5 (LT); 13.x.2017, 3 (LT); 10.xi.2017, 4 (LT); 2.xii.2017, 1 (LT).

**Remarks.** This species was frequently collected by LT in sand dunes.

**30. *Tetragonoderus quadrum* (Fabricius, 1792)**

**Material examined. Sajid Island**: Khotp, 16°52'27.9"N, 41°54'43.9"E, 9 m, 25.i.2017, 3 (PT); 18.ii.2017, 3 (PT). Khotp, 16°52'47.6"N, 41°54'39.7"E, 11 m, 18.ii.2017, 3 (PT). Sajid, 16°45'42.6"N, 41°59'56.0"E, 6 m, 10.xi.2017, 3 (PT); 3.xii.2017, 4 (LT).

**Literature records.** Baha and Jizan (Abdel-Dayem et al. 2019).

**General distribution.**AFR species, distributed in Sahel from Senegal to Horn of Africa, southern Arabian Peninsula (KSA, Oman).

**Remarks.** The listed specimens were collected by PT under canopies of *Ind. oblongifolia* in limestone areas and *Sen. alexandrina* sand dunes. Also, the species was collected by LT in sand dune. Identification by M.S. Abdel-Dayem.

**31. *Trichis pallida* Klug, 1832**

**Material examined. Farasan Island**: Farasan Reserve, 16°42'26.3"N, 42°03'59.4"E, 5 m, 4.i.2017, 1 (SW); 16.xii.2016, 1 (SW). **Sajid Island**: Sajid, 16°45'42.6"N, 41°59'56.0"E, 6 m, 17.ii.2017, 1 (LT); 12.viii.2017, 1 (LT); 13.x.2017, 1 (LT); 13.x.2017, 1 (SW).

**Literature records.** Jizan (Britton 1948; Mateu 1986).

**General distribution.** AFR_SAR species, occurring in east Africa (Ethiopia, Kenya, Somalia), northward and eastward to Egypt, Israel, Arabian Peninsula (KSA, United Arab Emirates), Iran, and Pakistan.

**Remarks.** This species was collected by SW on *Ind. oblongifolia* in salt marshes and *Sen. alexandrina* in sand dunes; and by LT in a sand dune. Identification by I. Rasool.

#### Superfamily Dytiscoidea

**Family Dytiscidae**

**32. *Eretes sticticus* (Linnaeus, 1767)**

**Material examined. Sajid Island**: Sajid, 16°45'42.6"N, 41°59'56.0"E, 6 m, 13.x.2017, 3 (LT).

**Literature records.** Asir, Eastern Province, Madinah, and Riyadh (Abdel-Dayem et al. 2017).

**General distribution.**COS species, distributed throughout the world.

**Remarks.** The listed specimens were collected by LT near a temporary freshwater pool in sandy area. Identification by M.S. Abdel-Dayem.

**33. *Hydroglyphus angularis* (Klug, 1834)**

**Material examined. Sajid Island**: Sajid, 16°45'42.6"N, 41°59'56.0"E, 6 m, 13.x.2017, 16 (LT).

**Literature records.** Jizan (Brancucci 1980).

**General distribution.**SAR species, distributed from the western Sahel to North Africa, east to Levant, Arabian Peninsula (KSA, Oman, Yemen (Socotra), Iran, and Pakistan.

**Remarks.** This species was collected by LT near a temporary freshwater pool in a sandy area. Identification by M.S. Abdel-Dayem.

**34. *Hydroglyphus geminus* (Fabricius, 1792)**

**Material examined. Sajid Island**: Sajid, 16°45'42.6"N, 41°59'56.0"E, 6 m, 13.x.2017, 3 (LT).

**Literature records.** Reported as *Guignotus pusillus* (Fabricius, 1781) from Eastern Province (Brancucci 1979).

**General distribution.** AUS_ORR_PAL_SAR species, widely distributed through the Sahara Desert from, from the Canary Island, east to Pakistan, India, Nepal, southeastern China, north to several countries in Europe, Central Asia, south to Australia.

**Remarks.** The species was collected by LT near a temporary freshwater pool in sandy area. Identification by M.S. Abdel-Dayem.

**35. *Hydroglyphus signatellus* (Klug, 1834)**

**Material examined. Sajid Island**: Sajid, 16°45'42.6"N, 41°59'56.0"E, 6 m, 13.x.2017, 1 (LT).

**Literature records.** Eastern Province and Riyadh (Abdel-Dayem et al. 2017).

**General distribution.** AFR_PAL_SAR species, distributed in West and East Africa, north to the entire of the Sahara Desert from Morocco east to Levant, Arabian Peninsula (KSA, Kuwait, Oman, United Arab Emirates, Yemen (Socotra)), Iran, Afghanistan, Pakistan, north to Eastern and Southern Europe, Central Asia.

**Remarks.** The single listed specimen was collected by LT near a temporary freshwater pool in sandy area. Identification by M.S. Abdel-Dayem.

#### Suborder Polyphaga

**Superfamily Bostrichoidea**

**Family Bostrichidae**

**36. *Acantholyctus cornifrons* (Lesne, 1898)**

**Material examined. Sajid Island**: Bridge, 16°51'27.4"N, 41°55'49.0"E, 2 m, 13.x.2017, 1 (SW). Sajid, 16°45'42.6"N, 41°59'56.0"E, 6 m, 26.i.2017, 1 (LT); 3.iv.2017, 4 (LT); 23.v.2017, 2 (LT); 28.vii.2017, 1 (LT); 13.x.2017, 38 (LT); 10.xi.2017, 9 (LT); 2.xii.2017, 12 (LT).

**Literature records.** Riyadh (Abdel-Dayem et al. 2017).

**General distribution.** AFR_SAR species, distributed in the western, eastern, and southern Afrotropical Region; through North Africa to Arabian Peninsula (KSA, Oman, United Arab Emirates, Yemen) and Iran.

**Remarks.** The specimens of this species were frequently collected from sand dunes by SW on *Sen. alexandrina* or by LT. Identification by H.H. Fadl.

**37. *Bostrychoplites zickeli* (Marseul, 1867)**

**Material examined. Sajid Island**: Sajid, 16°45'42.6"N, 41°59'56.0"E, 6 m, 25.i.2017, 1 (SW); 26.i.2017, 1 (LT); 5.iii.2017, 1 (LT); 3.iv.2017, 2 (LT); 23.v.2017, 2 (LT); 10.vi.2017, 1 (LT); 28.vii.2017, 1 (LT); 10.xi.2017, 2 (LT); 3.xii.2017, 2 (LT).

**General distribution.**SAR species, distributed across North Africa, and known from Yemen. New country record for KSA.

**Remarks.** The species was frequently collected from sand dunes by SW on *Sen. alexandrina* and by LT. Identification by H.H. Fadl.

**38. *Calopertha truncatula* (Ancey, 1881)**

**Material examined. Sajid Island**: Bridge, 16°51'27.4"N, 41°55'49.0"E, 2 m, 18.ii.2017, 1 (SW); Sajid, 16°45'42.6"N, 41°59'56.0"E, 6 m, 25.i.2017, 19 (LT); 26.i.2017, 4 (sw); 17.ii.2017, 4 (LT); 5.iii.2017, 236 (LT); 28.iv.2017, 5 (LT); 23.v.2017, 22 (LT); 10.vi.2017, 63 (LT); 28.vii.2017, 4 (LT); 28.vii.2017, 90 (LT); 12.viii.2017, 141 (LT); 5.ix.2017, 27 (LT); 13.x.2017, 20 (LT); 10.xi.2017, 5 (LT); 3.xii.2017, 4 (LT).

**General distribution.** AFR_SAR species, distributed in east Africa and southern Arabian Peninsula (Yemen), North Africa (Morocco, Egypt), and Iran to Pakistan. New country record for KSA.

**Remarks.** This species was abundant and frequently collected from sand dunes by SW on *Sen. alexandrina* and by LT. Identification by H.H. Fadl.

**39. *Enneadesmus forficula* (Fairmaire, 1883)**

**Material examined. Sajid Island**: Sajid, 16°45'42.6"N, 41°59'56.0"E, 6 m, 25.i.2017, 2 (LT); 5.iii.2017, 5 (LT); 4.iv.2017, 3 (LT); 23.v.2017, 5 (LT); 10.vi.2017, 5 (LT); 28.vii.2017, 3 (LT); 12.viii.2017, 10 (LT); 5.ix.2017, 1 (LT); 13.x.2017, 1 (LT); 10.xi.2017, 5 (LT); 3.xii.2017, 2 (LT).

**Literature records.** Makkah and Riyadh (Abdel-Dayem et al. 2017).

**General distribution.** AFR_SAR species, distributed in Horn of Africa (Eritrea, Ethiopia, Somalia); extending from Morocco eastwardly through Sahara to Levant, Arabian Peninsula (KSA, Oman, United Arab Emirates, Yemen), and from Iran to Pakistan.

**Remarks.** The listed specimens were frequently collected by LT from sand dunes. Identification by H.H. Fadl.

#### Family Dermestidae

**40. *Anthrenus flavipes* Leconte, 1854**

**Material examined. Sajid Island**: Khotp, 16°52'27.9"N, 41°54'43.9"E, 9 m, 27.i.2017, 1 (SW). Sajid, 16°45'42.6"N, 41°59'56.0"E, 6 m, 28.iv.2017, 1 (LT); 13.x.2017, 1 (SW).

**Literature records.** Riyadh (Abdel-Dayem et al. 2017).

**General distribution.**COS species distributed throughout the world.

**Remarks.** The species was collected by SW on *Sen. alexandrina* and LT in limestone area and sand dunes. Identification by M.S. Abdel-Dayem.

**41. *Attagenus obtusus* (Gyllenhal in Schönherr, 1808)**

**Material examined. Sajid Island**: Sajid, 16°45'42.6"N, 41°59'56.0"E, 6 m, 25.i.2017, 1 (LT).

**Literature records.**KSA is the only representative of the Arabian Peninsula that is listed within the distribution range of this species in the Catalogue of Palaearctic Coleoptera (Háva 2007), subsequently *A. obtusus* was excluded from the Arabian fauna by Háva (2012).

**General distribution.** PAL_SAR species, widely distributed through Sahara Desert, from the eastern Canary Islands, east across North Africa and Arabian Peninsula (KSA) to Iran; north to the southwestern Palaearctic region, in eastern, northern, southern, and western Europe.

**Remarks.** The single listed specimen was collected by LT in a sand dune. Identification by M.S. Abdel-Dayem.

**42. *Attagenus posticalis* Fairmaire, 1879**

**Material examined. Farasan Island**: Al-Kosar, 16°40'14.8"N, 42°08'54.9"E, 7 m, 25.i.2017, 3 (SW). **Sajid Island**: Khotp, 16°52'27.9"N, 41°54'43.9"E, 9 m, 6.ix.2017, 1 (SW). Sajid, 16°45'42.6"N, 41°59'56.0"E, 6 m, 25.i.2017, 7 (SW).

**Literature records.** Eastern Province and Riyadh (Abdel-Dayem et al. 2017).

**General distribution.** AFR_SAR species, distributed in Sahel and North Africa, east to Levant and Arabian Peninsula (KSA, Oman, Qatar, United Arab Emirates, Yemen (Socotra)), north to Spain.

**Remarks.** This species was frequently collected by SW on *Ind. oblongifolia* and *Sen. alexandrina* in limestone areas and sand dunes, respectively. Identification by M.S. Abdel-Dayem.

**43. *Attagenus* sp.**

**Material examined. Sajid Island**: Khotp, 16°52'47.6"N, 41°54'39.7"E, 11 m, 26.i.2017, 2 (LT). Sajid, 16°45'42.6"N, 41°59'56.0"E, 6 m, 3.xii.2017, 1 (SW).

**Remarks.** The listed specimens were collected by LT in limestone and by SW on *Sen. alexandrina* from sand dunes.

**44. *Phradonoma* sp.**

**Material examined. Sajid Island**: Sajid, 16°45'42.6"N, 41°59'56.0"E, 6 m, 10.vi.2017, 1 (LT); 13.x.2017, 2 (LT).

**Remarks.** These listed specimens were collected by LT from sand dunes.

**45. *Phradonoma tricolor* (Arrow, 1915)**

**Material examined. Sajid Island**: Khotp, 16°52'47.6"N, 41°54'39.7"E, 11 m, 25.i.2017, 1 (SW); 18.ii.2017, 3 (SW). Sajid, 16°45'42.6"N, 41°59'56.0"E, 6 m, 25.i.2017, 1 (LT); 28.iv.2017, 1 (LT).

**Literature records.** The species was described from Yemen and the probability of its occurrence in KSA has been mentioned by Mroczkowski (1979); however, no specimens have been previously examined from KSA before this study.

**General distribution.** ORR_SAR species, cited from Arabian Peninsula (KSA, Oman, Yemen) and India.

**Remarks.** The listed specimens were collected by SW on *Ind. oblongifolia* and *Sen. alexandrina* in limestone areas and by LT in sand dunes. Identification by A.A. Elgharbawy.

#### Family Ptinidae

**46. *Anobium* sp.**

**Material examined. Sajid Island**: Sajid, 16°45'42.6"N, 41°59'56.0"E, 6 m, 10.xi.2017, 2 (LT); 13.x.2017, 1 (LT).

**Remarks.** The listed specimens were collected by LT from sand dunes.

**47. *Dignomus mesopotamicus* (Pic, 1894)**

**Material examined. Sajid Island**: Khotp, 16°52'27.9"N, 41°54'43.9"E, 9 m, 25.i.2017, 1 (SW); 5.iii.2017, 3 (SW). Khotp, 16°52'47.6"N, 41°54'39.7"E, 11 m, 5.iii.2017, 2 (SW). Sajid, 16°45'42.6"N, 41°59'56.0"E, 6 m, 17.ii.2017, 1 (LT); 5.iii.2017, 2 (LT).

**Literature records.** The information on Saudi local distribution is not available.

**General distribution.**SAR species, distributed in the Arabian Peninsula (KSA, Yemen (Socotra)), Iraq and Iran.

**Remarks.** The specimens of this species were collected by SW in limestone areas and LT in sand dunes. Identification by H.H. Fadl.

**48. *Lasioderma serricorne* (Fabricius, 1792)**

**Material examined. Sajid Island**: Sajid, 16°45'42.6"N, 41°59'56.0"E, 6 m, 13.x.2017, 3 (LT).

**Literature records.** Makkah (Toskina 1998).

**General distribution.**COS species, distributed throughout the world.

**Remarks.** This species was collected by LT from sand dunes. Identification by H.H. Fadl.

**49. *Stegobium paniceum* (Linnaeus, 1758)**

**Material examined. Sajid Island**: Sajid, 16°45'42.6"N, 41°59'56.0"E, 6 m, 25.i.2017, 2 (LT); 17.ii.2017, 9 (LT); 5.iii.2017, 23 (LT); 10.iv.2017, 9 (LT); 6.ix.2017, 8 (LT); 13.x.2017, 1 (LT); 10.xi.2017, 3 (LT); 2.xii.2017, 2 (LT).

**Literature records.** Previous specific locality information for Saudi Arabian specimens are not available to us.

**General distribution.**COS species, distributed throughout the world.

**Remarks.** The listed specimens were frequently collected by LT from sand dunes. Identification by H.H. Fadl.

#### Superfamily Buprestoidea

**Family Buprestidae**

**50. *Agrilus purpuratus* (Klug, 1829)**

**Material examined. Farasan Island**: Al-Huseis, 16°45'20.8"N, 42°03'59.6"E, 7 m, 26.i.2017, 1 (SW); 23.v.2017, 1 (SW). Al-Kosar, 16°40'14.8"N, 42°08'54.9"E, 7 m, 25.i.2017, 1 (SW); 23.v.2017, 1 (SW). Farasan Reserve, 16°42'26.5"N, 42°04'19.2"E, 7 m, 26.i.2017, 1 (SW); 5.iii.2017, 1 (SW). **Sajid Island**: Sajid, 16°45'42.6"N, 41°59'56.0"E, 6 m, 25.i.2017, 3 (LT); 6.iii.2017, 2 (SW); 23.v.2017, 1 (LT); 28.vii.2017, 1 (LT).

**Literature records.** Asir (Bílý 1982).

**General distribution.** AFR_SAR species, distributed throughout North Africa, eastwardly from Levant and Arabian Peninsula (KSA, Yemen) to Iran, southwards to the Horn of Africa (Somalia).

**Remarks.** This species was collected by SW on *Sua. monoica* in salt marshes, *Ind. oblongifolia* in limestone areas, and *Sen. alexandrina* in sand dunes. Also, it was collected by LT from sand dunes and SW in coastal sandy areas. Identification by D. Baiocchi and G. Magnani.

**51. *Anthaxia congregata* (Klug, 1829)**

**Material examined. Farasan Island**: Farasan Reserve, 16°42'26.5"N, 42°04'19.2"E, 7 m, 5.ix.2017, 1 (SW).

**Literature records.** Asir and Riyadh (Bílý 1982).

**General distribution.** AFR_SAR species, distributed from Morocco to Egypt across North Africa, east to Levant, Arabian Peninsula (KSA) and Iran.

**Remarks.** This unique listed specimen was collected from SW on branches of *Sua. monoica* in a salt marsh. Identification by D. Baiocchi and G. Magnani.

**52. *Lampetis argentata* (Mannerheim, 1837)**

**Material examined. Farasan Island**: Al-Kosar, 16°40'14.8"N, 42°08'54.9"E, 7 m, 7.i.2017, 1 (SW). Sajid, 16°45'42.6"N, 41°59'56.0"E, 6 m, 5.ii.2017, 1 (LT); 4.iv.2017, 1 (LT); 23.v.2017, 1 (LT); 26.vi.2017, 1 (LT); 2.xii.2017, 1 (LT).

**Literature records.** Asir, Eastern Province, and Riyadh (Bílý 1982).

**General distribution.** PAL_SAR species, distributed in south Caucasus, Anatolia, and Central Asia, south to Arabia Peninsula (KSA), Iraq, Iran, and Afghanistan.

**Remarks.** The species was collected by SW on *Lim. axillare* and *Sen. alexandrina*, and by LT in sand dunes. Identification by D. Baiocchi and G. Magnani.

**53. *Sphenoptera gossypicida* Obenberger, 1927**

**Material examined. Farasan Island**: Al-Huseis, 16°45'20.8"N, 42°03'59.6"E, 7 m, 5.xi.2017, 2 (SW). Al-Kosar, 16°40'14.8"N, 42°08'54.9"E, 7 m, 5.ix.2017, 1 (SW); 13.x.2017, 1 (SW). Farasan Reserve, 16°42'26.5"N, 42°04'19.2"E, 7 m, 5.ix.2017, 1 (SW). **Sajid Island**: Khotp, 16°52'27.9"N, 41°54'43.9"E, 9 m, 5.ix.2017, 1 (SW); 13.x.2017, 2 (SW); 2.xii.2017, 1 (SW). Khotp, 16°52'47.6"N, 41°54'39.7"E, 11 m, 26.i.2017, 1 (SW); 6.iii.2017, 2 (SW); 6.ix.2017, 2 (SW); 13.x.2017, 3 (SW). Sajid, 16°45'42.6"N, 41°59'56.0"E, 6 m, 26.i.2017, 1 (SW); 17.ii.2017, 1 (LT); 5.iii.2017, 2 (SW); 5.iii.2017, 1 (LT); 5.ix.2017, 1 (LT); 5.ix.2017, 1 (SW); 13.x.2017, 1 (SW); 13.x.2017, 1 (LT); 3.xii.2017, 1 (SW).

**Literature records.** Jizan (Bílý 1982).

**General distribution.**SAR species known only from Egypt (including Sinai), Sudan, and Arabian Peninsula (KSA).

**Remarks.** This species was collected by SW branches on *Ind. oblongifolia*, *Lim. axillare*, and *Sua. monoica* in limestone areas, sand dunes and salt marshes, respectively. Also, this species was collected by LT in sand dunes and SW in coastal sandy areas. Identification by D. Baiocchi and G. Magnani.

#### Superfamily Chrysomeloidea

**Family Cerambycidae**

**54. *Cantharocnemis spondyloides* Audinet-Serville, 1832**

**Material examined. Sajid Island**: Bridge, 16°51'27.4"N, 41°55'49.0"E, 2 m, 23.v.2017, 2 (SW). Sajid, 16°45'42.6"N, 41°59'56.0"E, 6 m, 17.ii.2017, 2 (LT); 5.iii.2017, 1 (LT); 10.xi.2017, 3 (LT).

**Literature records.** Jizan and Makkah (Holzschuh 1993).

**General distribution.** AFR_MAD species, widely distributed in tropical Africa, Madagascar, Egypt, and southern Arabian Peninsula (KSA, Oman, Yemen (Socotra)).

**Remarks.** The listed specimens were frequently collected in sand dunes from *Dic. foveolatum* and *Ind. oblongifolia*. Identification by P. Rapuzzi.

**55. *Eunidia breuningae* Villiers, 1951**

**Material examined. Sajid Island**: Khotp, 16°52'27.9"N, 41°54'43.9"E, 9 m, 3.xii.2017, 2 (SW). Sajid, 16°45'42.6"N, 41°59'56.0"E, 6 m, 10.x.2017, 2 (LT).

**Literature records.** Asir, Jizan and Makkah (Holzschuh and Téocchi 1991).

**General distribution.**AFR species, distributed in Sahel part of Africa (Chad, Djibouti, Niger, Senegal), north to Egypt, east to southern Arabian Peninsula (KSA, Oman, Yemen).

**Remarks.** The species was collected by LT and SW in limestone areas and sand dunes. Identification by P. Rapuzzi.

#### Family Chrysomelidae

**56. *Acolastus wittmeri* (Lopatin, 1979)**

**Material examined. Farasan Island**: Al-Huseis, 16°45'20.8"N, 42°03'59.6"E, 7 m, 26.i.2017, 1 (SW). Farasan Reserve, 16°42'26.3"N, 42°03'59.4"E, 5 m, 17.ii.2017, 1 (SW); 5.ix.2017, 1 (SW). Farasan Reserve, 16°42'26.5"N, 42°04'19.2"E, 7 m, 26.i.2017, 1 (SW); 5.ix.2017, 1 (SW). **Sajid Island**: Khotp, 16°52'27.9"N, 41°54'43.9"E, 9 m, 5.ix.2017, 1 (SW); 2.xii.2017, 1 (SW). Khotp, 16°52'47.6"N, 41°54'39.7"E, 11 m, 5.iii.2017, 1 (SW); 5.ix.2017, 2 (SW). Sajid, 16°45'42.6"N, 41°59'56.0"E, 6 m, 5.iii.2017, 2 (SW); 5.ix.2017, 1 (SW); 10.x.2017, 2 (SW).

**Literature records.** This species was described Asir and Riyadh (Lopatin 1979).

**General distribution.** END_AR species, so far known only from KSA and Yemen.

**Remarks.** The listed specimens were collected by SW on *Ind. oblongifolia*, *Sen. alexandrina*, and *Sua. monoica* in limestone areas, sand dunes, and salt marshes, respectively, and in coastal sandy areas. Identification by A. El-Torkey and J. Bezděk.

**57. *Angulaphthona latipennis* (Pic, 1921)**

**Material examined. Sajid Island**: Khotp, 16°52'47.6"N, 41°54'39.7"E, 11 m, 28.iv.2017, 1 (SW); 2.xii.2017, 1 (SW). Sajid, 16°45'42.6"N, 41°59'56.0"E, 6 m, 7.i.2017, 1 (SW); 23.v.2017, 1 (SW).

**Literature records.** Jizan (Medvedev 1996).

**General distribution.**AFR species, distributed through Sahel part of Africa, from Niger east to Sudan and the Horn of Africa (Eritrea, Somalia); Congo, south and southeast of Africa; southern Arabian Peninsula (KSA, Yemen).

**Remarks.** The listed specimens were collected by SW on *Ind. oblongifolia* and *Sen. alexandrina* in limestone areas and sand dunes, respectively. Identification by A. El-Torkey and J. Bezděk.

**58. *Bruchidius medaniensis* (Decelle, 1982)**

**Material examined. Sajid Island**: Bridge, 16°51'27.4"N, 41°55'49.0"E, 2 m, 5.ix.2017, 2 (SW). Khotp, 16°52'27.9"N, 41°54'43.9"E, 9 m, 5.iii.2017, 1 (SW). Khotp, 16°52'47.6"N, 41°54'39.7"E, 11 m, 5.iii.2017, 1 (SW). Sajid, 16°45'42.6"N, 41°59'56.0"E, 6 m, 23.v.2017, 1 (SW).

**Literature records.** This species was described by Decelle (1982) from Sudan, and 28 years later, Delobel and Le Ru (2010) reported it from Kenya. The first record of this species from the Arabian Peninsula was reported by Delobel (2011) from United Arab Emirates. In the Catalogue of Palaearctic Coleoptera, KSA and Yemen is listed among the distribution range of the species (Anton 2010). No previously published specific KSA locality records for this species are known to us.

**General distribution.**AFR species, distributed in eastern Africa (Kenya, Sudan) and southern Arabian Peninsula (United Arab Emirates).

**Remarks.** The specimens were collected by SW on *Ind. oblongifolia* in limestone areas and sand dunes, *Dic. foveolatum* in sand dunes, and *Sen. alexandrina* in limestone areas. Identification by A. El-Torkey.

**59. *Bruchidius* sp.**

**Material examined. Sajid Island**: Khotp, 16°52'47.6"N, 41°54'39.7"E, 11 m, 5.iii.2017, 1 (SW). Sajid, 16°45'42.6"N, 41°59'56.0"E, 6 m, 23.v.2017, 2 (SW); 2.xii.2017, 3 (SW).

**Remarks.** The listed specimens were collected by SW on *Ind. oblongifolia* in limestone areas and *Sen. alexandrina* in sand dunes.

**60. *Bruchus* sp.**

**Material examined. Sajid Island**: Khotp, 16°52'27.9"N, 41°54'43.9"E, 9 m, 10.vi.2017, 3 (SW); 2.xii.2017, 1 (SW). Khotp, 16°52'47.6"N, 41°54'39.7"E, 11 m, 2.xii.2017, 1 (SW). Sajid, 16°45'42.6"N, 41°59'56.0"E, 6 m, 28.iv.2017, 4 (LT); 2.xii.2017, 2 (LT).

**Remarks.** The specimens of this species were collected by SW on *Sen. alexandrina* in sand dunes and by LT in limestone areas.

**61. *Callosobruchus* sp.**

**Material examined. Sajid Island**: Sajid, 16°45'42.6"N, 41°59'56.0"E, 6 m, 23.v.2017, 1 (SW).

**Remarks.** The species was collected by SW on *Sen. alexandrina* in sand dunes.

**62. Caryedon cf. yemenensis Decelle, 1979**

**Material examined. Sajid Island**: Khotp, 16°52'27.9"N, 41°54'43.9"E, 9 m, 5.iii.2017, 1 (SW); 6.ix.2017, 3 (SW). Khotp, 16°52'47.6"N, 41°54'39.7"E, 11 m, 2.xii.2017, 1 (SW). Sajid, 16°45'42.6"N, 41°59'56.0"E, 6 m, 25.i.2017, 1 (SW); 25.i.2017, 3 (LT); 17.ii.2017, 4 (LT); 5.iii.2017, 6 (LT); 28.iv.2017, 1 (LT); 13.x.2017, 5 (LT); 13.x.2017, 2 (SW); 10.xi.2017, 16 (LT); 2.xii.2017, 4 (LT); 2.xii.2017, 1 (SW).

**Literature records.** Makkah and Riyadh (Decelle 1979).

**General distribution.**SAR species, distributed in Egypt, Levant, Arabian Peninsula (KSA, Oman, United Arab Emirates, Yemen), Iran, and Pakistan.

**Remarks.** The listed specimens were collected on *Ind. oblongifolia* and *Sen. alexandrina* in limestone areas and sand dunes, respectively. Also, the species was collected by LT in sand dunes. Identification by A. El-Torkey.

**63. *Caryedon gonagra* (Fabricius, 1798)**

**Material examined. Sajid Island**: Sajid, 16°45'42.6"N, 41°59'56.0"E, 6 m, 28.iv.2017, 2 (LT); 10.vi.2017, 5 (LT); 13.x.2017, 5 (LT).

**Literature records.** Although most of the Arabian Peninsula countries are listed within the distribution range of this species (Anton 2010), none of the countries, except the United Arab Emirates are included in the distribution of the species as documented by Delobel (2011). We know of no previous published specific KSA records for this species.

**General distribution.** AUS_ORR_SAR species, distributed from Egypt, Levant, Arabian Peninsula (KSA, Kuwait, Oman, United Arab Emirates, Yemen (Socotra)) and Iran east to Pakistan, India and southeast Asia, and south to Australia. Widely distributed in the tropics of the Old World

**Remarks.** The specimens of this species were collected by LT in sand dunes. Identification by A. El-Torkey.

**64. *Colasposoma* sp.**

**Material examined. Sajid Island**: Khotp, 16°52'47.6"N, 41°54'39.7"E, 11 m, 25.i.2017, 1 (SW). Sajid, 16°45'42.6"N, 41°59'56.0"E, 6 m, 12.ii.2017, 3 (SW); 17.ii.2017, 3 (LT); 28.iv.2017, 2 (SW); 23.v.2017, 1 (SW); 10.vi.2017, 1 (SW).

**Remarks.** The listed specimens were collected by SW on *Idn. oblongifolia* and *Sen. alexandrina* in limestone areas and sand dunes, respectively.

**65. *Hermaeophaga ruficollis* (Lucas,1847)**

**Material examined. Sajid Island**: Khotp, 16°52'47.6"N, 41°54'39.7"E, 11 m, 17.ii.2017, 1 (SW).

**Literature records.** Reported as *Orthocrepis ruficollis* from Jizan (Medvedev 1996).

**General distribution.** AFR_ORR_PAL_SAR species, distributed in whole North Africa and Sahel; eastward through Levant, Arabian Peninsula (KSA, Oman, United Arab Emirates, Yemen (Socotra)) and Iran to Pakistan, India, and Nepal; northward to southern Europe and central Asia.

**Remarks.** The single listed specimen of this species was collected by SW on *Sen. alexandrina* in a sand dune. Identification by A. El-Torkey and J. Bezděk.

**66. *Heteraspis vicina* (Harold, 1877)**

**Material examined. Sajid Island**: Khotp, 16°52'27.9"N, 41°54'43.9"E, 9 m, 5.iii.2017, 1 (SW).

**Literature records.** Reported from Asir, Baha, and Makkah as *Scelodonta vicina* (Daccordi 1979; Medvedev 1996).

**General distribution.**AFR species, distributed in Central Africa (Congo), East Africa (Ethiopia, Mozambique, Tanzania) and southern Arabian Peninsula (KSA, Oman, Yemen).

**Remarks.** The listed unique specimen was collected by SW on *Ind. oblongifolia* in a limestone area. Identification by A. El-Torkey and J. Bezděk.

**67. *Macrocoma* sp.**

**Material examined. Sajid Island**: Khotp, 16°52'47.6"N, 41°54'39.7"E, 11 m, 5.iii.2017, 1 (SW). Sajid, 16°45'42.6"N, 41°59'56.0"E, 6 m, 25.i.2017, 2 (SW).

**Remarks.** The specimens were collected by SW on *Ind. oblongifolia* and *Sen. alexandrina* in limestone areas and sand dunes, respectively.

**68. *Medythia* sp.**

**Material examined. Sajid Island**: Khotp, 16°52'27.9"N, 41°54'43.9"E, 9 m, 4.ii.2017, 2 (SW).

**Remarks.** The listed specimens were collected by SW on *Ind. oblongifolia* in limestone area.

**69. *Melixanthus melanocephalus* (Suffrian, 1857)**

**Material examined. Sajid Island**: Khotp, 16°52'27.9"N, 41°54'43.9"E, 9 m, 2.xii.2017, 1 (SW).

**Literature records.** Reported as *Melixanthus jordanicus* Lopatin, 1979 from Asir, Jizan, Makkah, and Riyadh (Lopatin 1979, 1983).

**General distribution.**SAR species, distributed from North Africa (Egypt, Libya) to Levant, Arabian Peninsula (KSA, United Arab Emirates, Yemen (Socotra)), Iran, and Armenia.

**Remarks.** The listed single specimen was collected by SW on *Ind. oblongifolia* in a limestone area. Identification by A. El-Torkey and J. Bezděk.

**70. *Paraclytra sennariensis* (Lacordaire, 1848)**

**Material examined. Farasan Island**: Al-Huseis, 16°45'20.8"N, 42°03'59.6"E, 7 m, 10.vi.2017, 1 (SW); 28.vii.2017, 1 (SW); 6.ix.2017, 1 (SW); 10.x.2017, 1 (SW). Al-Kosar, 16°40'14.8"N, 42°08'54.9"E, 7 m, 7.i.2017, 2 (SW); 10.vi.2017, 1 (SW); 6.ix.2017, 1 (SW); 10.x.2017, 1 (SW). Farasan Reserve, 16°42'26.3"N, 42°03'59.4"E, 5 m, 23.v.2017, 1 (SW); Farasan Reserve, 16°42'26.5"N, 42°04'19.2"E, 7 m, 23.v.2017, 1 (SW). **Sajid Island**: Bridge, 16°51'27.4"N, 41°55'49.0"E, 2 m, 18.ii.2017, 1 (SW); 10.xi.2017, 2 (SW). Khotp, 16°52'27.9"N, 41°54'43.9"E, 9 m, 5.iii.2017, 1 (SW); 10.vi.2017, 1 (SW); 6.ix.2017, 1 (SW). Khotp, 16°52'47.6"N, 41°54'39.7"E, 11 m, 5.iii.2017, 1 (SW); 10.vi.2017, 1 (SW); 6.ix.2017, 1 (SW); 10.xi.2017, 3 (SW). Sajid, 16°45'42.6"N, 41°59'56.0"E, 6 m, 26.i.2017, 1 (LT); 5.iii.2017, 4 (SW); 10.vi.2017, 2 (SW); 6.ix.2017, 2 (SW); 10.x.2017, 3 (SW); 10.xi.2017, 3 (SW); 3.xii.2017, 3 (SW).

**Literature records.** This species was reported as *P. signata sennariensis* from Jizan, Makkah, and Riyadh (Medvedev 1993). *Paraclytra signata sennariensis* was elevated to specific rank by Bezděk and Kantner (2010).

**General distribution.** AFR_SAR species, distributed through Sahel and the Horn of Africa, east to western Arabian Peninsula (KSA, Yemen), and north to Egypt and Syria.

**Remarks.** This species was collected by SW on *Lim. axillare* and *Sen. alexandrina* in sand dunes, and on *Ind. oblongifolia* and *Sua. monoica* in limestone areas and salt marshes, respectively. Also, it was collected by LT and SW in sand dunes and coastal sandy areas, respectively. Identification by A. El-Torkey and J. Bezděk.

**71. *Phyllotreta* sp.**

**Material examined. Sajid Island**: Sajid, 16°45'42.6"N, 41°59'56.0"E, 6 m, 3.xii.2017, 1 (SW).

**Remarks.** The single listed specimen was collected by SW on *Sen. alexandrina* in a sand dune.

**72. *Podagrica puncticollis* Wise, 1902**

**Material examined. Farasan Island**: Farasan Reserve, 16°42'26.3"N, 42°03'59.4"E, 5 m, 23.v.2017, 1 (SW). **Sajid Island**: Bridge, 16°51'27.4"N, 41°55'49.0"E, 2 m, 28.vii.2017, 1 (SW). Khotp, 16°52'27.9"N, 41°54'43.9"E, 9 m, 28.vii.2017, 2 (SW). Khotp, 16°52'47.6"N, 41°54'39.7"E, 11 m, 28.vii.2017, 1 (SW). Sajid, 16°45'42.6"N, 41°59'56.0"E, 6 m, 10.vi.2017, 1 (LT); 28.vii.2017, 3 (SW); 10.x.2017, 2 (SW); 3.xii.2017, 14 (SW).

**Literature records.** Jizan and Makkah (Abu-Thuraya 1982).

**General distribution.**AFR species, distributed through Sahel and East Africa, north to Egypt, and east to southern Arabian Peninsula (KSA, Oman, Yemen (Socotra)).

**Remarks.** The specimens of this species were collected in limestone areas, salt marshes and sand dunes by SW on *Ind. oblongifolia*, *Sua. monoica*, and *Sen. alexandrina*, respectively. Identification by A. El-Torkey and J. Bezděk.

#### Superfamily Cleroidea

**Family Cleridae**

**73. *Opilo longipilis* Fairmaire, 1892**

**Material examined. Sajid Island**: Sajid, 16°45'42.6"N, 41°59'56.0"E, 6 m, 13.x.2017, 1 (LT).

**Literature records.** Baha, Hail, Madinah, Makkah, and Riyadh (Menier 1986).

**General distribution.**SAR species, only known from Algeria and Arabian Peninsula (KSA).

**Remarks.** The unique specimen of this species was collected by LT in a sand dune. Identification by H.H. Fadl.

**74. *Phloiocopus tricolor* Guérin-Méneville, 1835**

**Material examined. Sajid Island**: Sajid, 16°45'42.6"N, 41°59'56.0"E, 6 m, 10.xi.2017, 1 (LT).

**Literature records.** Jizan, Makkah and Qaseem (Menier 1986).

**General distribution.** Probably AFR species, known from Senegal, southwestern Arabian Peninsula (KSA, Yemen), and Syria.

**Remarks.** The single listed specimen was collected by LT in a sand dune. Identification by H.H. Fadl.

**75. *Tillodenops plagiatus* (Fairmaire, 1892)**

**Material examined. Sajid Island**: Sajid, 16°45'42.6"N, 41°59'56.0"E, 6 m, 6.ix.2017, 1 (LT); 13.x.2017, 1 (LT); 2.xii.2017, 1 (LT).

**Literature records.** Asir, Baha, Jizan, Madinah, Makkah, and Riyadh (Abdel-Dayem et al. 2017).

**General distribution.** AFR_SAR species, distributed in West (Mauritania, Senegal) and East Africa (Kenya, Somalia, Sudan, Tanzania), north to Libya, and east to Arabian Peninsula (KSA, Oman, United Arab Emirates, Yemen) and Iran.

**Remarks.** The species was collected by LT in sand dunes. Identification by H.H. Fadl.

**76. *Wittmeridecus mediozonatus* (Fairmaire, 1892)**

**Material examined. Sajid Island**: Sajid, 16°45'42.6"N, 41°59'56.0"E, 6 m, 28.vii.2017, 1 (LT); 10.xi.2017, 1 (LT); 3.xii.2017, 1 (LT).

**Literature records.** Asir, Madinah, Makkah, Riyadh (Abdel-Dayem et al. 2017).

**General distribution.**SAR species, distributed through entire North Africa east to Levant and Arabian Peninsula (KSA, Oman, United Arab Emirates, Yemen), north to southern Europe (Italy, Spain).

**Remarks.** The specimens of this species were collected by LT in sand dunes. Identification by H.H. Fadl.

#### Family Melyridae

**77. *Melyris bicolor* Fabricius, 1801**

**Material examined. Farasan Island**: Al-Kosar, 16°40'14.8"N, 42°08'54.9"E, 7 m, 25.i.2017, 7 (SW). Farasan Reserve, 16°42'26.3"N, 42°03'59.4"E, 5 m, 17.ii.2017, 1 (SW). **Sajid Island**: Khotp, 16°52'47.6"N, 41°54'39.7"E, 11 m, 25.i.2017, 7 (SW). Sajid, 16°45'42.6"N, 41°59'56.0"E, 6 m, 26.i.2017, 2 (LT); 3.xii.2017, 1 (SW).

**Literature records**. Previous specific locality information for KSA records was not available to us.

**General distribution.**SAR species, reported from Egypt, Syria, and Arabian Peninsula (KSA).

**Remarks.** The listed specimens were collected by SW on *Ind. oblongifolia*, *Sua. monoica*, and *Sen. alexandrina* in limestone areas, salt marshes, and sand dunes, respectively. Also, the species was collected by LT in a sand dune. Identification by H.H. Fadl.

#### Superfamily Cucujoidea

**Family Coccinellidae**

**78. *Bulaea lividula bocandei* Mulsant, 1850**

**Material examined. Sajid Island**: Khotp, 16°52'27.9"N, 41°54'43.9"E, 9 m, 10.vi.2017, 3 (SW); 10.x.2017, 3 (SW). Khotp, 16°52'47.6"N, 41°54'39.7"E, 11 m, 25.i.2017, 9 (SW).

**Literature records.** Eastern Province and Riyadh (Abdel-Dayem et al. 2017).

**General distribution.** AFR_ORR_SAR species, distributed through North Africa, east to Levant, Arabian Peninsula (KSA, United Arab Emirates, Yemen), Iran, Pakistan, and India, also recorded from Eretria.

**Remarks.** The species was frequently collected by SW on *Aer. javanica*, *Abu. pannosum*, and *Cap. sinaica* in limestone areas. Identification by A.N. Al Ansi.

**79. *Cheilomenes propinqua vicina* (Mulsant, 1850)**

**Material examined. Sajid Island**: Sajid, 16°45'42.6"N, 41°59'56.0"E, 6 m, 27.i.2017, 5 (SW).

**Literature records.** Asir (Fürsch 1979).

**General distribution.** AFR_SAR species, distributed in Sahel and East Africa (Kenya), north through North Africa, east to Levant and Arabian Peninsula (KSA, Yemen (Socotra)).

**Remarks.** The listed specimens were collected by SW on *Sen. alexandrina* from sand dunes. Identification by A.N. Al Ansi.

**80. *Henosepilachna elaterii orientalis* K. Zimmermann, 1939**

**Material examined. Farasan Island**: Al-Huseis, 16°45'20.8"N, 42°03'59.6"E, 7 m, 27.iii.2017, 1 (SW). Al-Kosar, 16°40'14.8"N, 42°08'54.9"E, 7 m, 27.i.2017, 1 (SW). Farasan Reserve, 16°42'26.3"N, 42°03'59.4"E, 5 m, 27.i.2017, 1 (SW). Farasan Reserve, 16°42'26.5"N, 42°04'19.2"E, 7 m, 27.iii.2017, 1 (SW). **Sajid Island**: Khotp, 16°52'27.9"N, 41°54'43.9"E, 9 m, 10.x.2017, 2 (SW). Khotp, 16°52'47.6"N, 41°54'39.7"E, 11 m, 10.x.2017, 2 (SW). Sajid, 16°45'42.6"N, 41°59'56.0"E, 6 m, 10.x.2017, 2 (SW); 13.x.2017, 1 (SW).

**Literature records.** Riyadh (Fürsch 1979).

**General distribution.** AFR_SAR species, distributed in tropical Africa (South Africa), Sahel, and North Africa (Egypt, Libya), east from Levant, Arabian Peninsula (KSA, Kuwait, Oman, Yemen), and Iran to Pakistan.

**Remarks.** This species was collected by SW on *Ind. oblongifolia*, *Sen. alexandrina*, and *Sua. monoica* in limestone areas, sand dunes, and salt marshes. Also, it was collected in coastal sandy areas by SW. Identification by A.N. Al Ansi.

**81. *Hyperaspis vinciguerrae* Capra, 1929**

**Material examined. Sajid Island**: Bridge, 16°51'27.4"N, 41°55'49.0"E, 2 m, 18.ii.2017, 1 (SW); 27.iv.2017, 5 (SW). Khotp, 16°52'47.6"N, 41°54'39.7"E, 11 m, 5.iii.2017, 1 (SW). Sajid, 16°45'42.6"N, 41°59'56.0"E, 6 m, 3.xii.2017, 2 (SW).

**Literature records.** Riyadh (Abdel-Dayem et al. 2017).

**General distribution.** AFR_SAR species distributed in West Africa (Gambia, Senegal) and North Africa (Libya), east in Arabian Peninsula (KSA, United Arab Emirates, Yemen) and Iran.

**Remarks.** The listed specimens were collected by SW on *Ind. oblongifolia* in limestone areas and sand dunes, and on *Aer. javanica* and *Sen. alexandrina* from sand dunes. Identification by A.N. Al Ansi.

**82. *Nephus arcuatus* Kapur, 1959**

**Material examined. Sajid Island**: Khotp, 16°52'27.9"N, 41°54'43.9"E, 9 m, 5.iii.2017, 1 (SW). Khotp, 16°52'47.6"N, 41°54'39.7"E, 11 m, 5.iii.2017, 1 (SW).

**Literature records.** Asir and Riyadh (Abdel-Dayem et al. 2017).

**General distribution.** AFR_SAR species, distributed in West Africa (Togo), Arabian Peninsula (KSA, United Arab Emirates, Yemen), and Iran.

**Remarks.** The species was collected by SW on *Ind. oblongifolia* in limestone areas. Identification by A.N. Al Ansi.

**83. *Parexochomus nigripennis* (Erichson, 1843)**

**Material examined. Farasan Island**: Al-Huseis, 16°45'20.8"N, 42°03'59.6"E, 7 m, 17.ii.2017, 3 (SW); 23.v.2017, 1 (SW). Al-Kosar, 16°40'14.8"N, 42°08'54.9"E, 7 m, 17.ii.2017, 2 (SW); 23.v.2017, 1 (SW). **Sajid Island**: Bridge, 16°51'27.4"N, 41°55'49.0"E, 2 m, 17.ii.2017, 3 (SW); 27.iv.2017, 6 (SW). Khotp, 16°52'27.9"N, 41°54'43.9"E, 9 m, 18.ii.2017, 12 (SW); 5.iii.2017, 15 (SW). Khotp, 16°52'47.6"N, 41°54'39.7"E, 11 m, 17.ii.2017, 6 (SW); 5.iii.2017, 4 (SW); 23.v.2017, 1 (SW). Sajid, 16°45'42.6"N, 41°59'56.0"E, 6 m, 17.ii.2017, 5 (SW).

**Literature records.** The species was recorded as *Exochomus nigreipennis* from Riyadh (Fürsch 1979).

**General distribution.** ORR_SAR species, distributed throughout North Africa, east from Levant, Arabian Peninsula (KSA, United Arab Emirates) and Iran to India, north to southern Europe (Italy, Malta, Portugal, Spain).

**Remarks.** This species was collected by SW on *Com. gileadensis* and *Sal. persica* in coastal sandy areas, *Ind. oblongifolia* in limestone areas, and *Aer. javanica*, *Abu. pannosum*, and *Sen. alexandrina* from sand dunes. Identification by A.N. Al Ansi.

**84. *Pharoscymnus numidicus* (Pic, 1900)**

**Material examined. Sajid Island**: Khotp, 16°52'47.6"N, 41°54'39.7"E, 11 m, 17.ii.2017, 1 (SW).

**Literature records.** Asir and Riyadh (Fürsch 1979).

**General distribution.**SAR species, distributed throughout North Africa, northern of Niger and Sudan, eastward to Levant and Arabian Peninsula (KSA).

**Remarks.** The unique listed specimen was collected by SW on *Ind. oblongifolia* in a limestone area. Identification by A.N. Al Ansi.

**85. *Scymnus nubilus* Mulsant, 1850**

**Material examined. Sajid Island**: Khotp, 16°52'47.6"N, 41°54'39.7"E, 11 m, 28.i.2017, 8 (SW).

**Literature records.** Asir and Riyadh (Abdel-Dayem et al. 2017).

**General distribution.**SCO species, widely distributed in the South, Southwest and East Asia, East and North Africa, southern Europe, and Australia.

**Remarks.** The specimens of this species were collected by SW on *Ind. oblongifolia* in limestone areas. Identification by A.N. Al Ansi.

**86. *Scymnus scapuliferus* Mulsant, 1850**

**Material examined. Sajid Island**: Al-Kosar, 16°40'14.8"N, 42°08'54.9"E, 7 m, 18.ii.2017, 1 (SW). Khotp, 16°52'27.9"N, 41°54'43.9"E, 9 m, 10.vi.2017, 2 (SW). Khotp, 16°52'47.6"N, 41°54'39.7"E, 11 m, 17.ii.2017, 2 (SW); 5.iii.2017, 2 (SW).

**General distribution.**AFR species, distributed in West and Central Africa, and cited from Yemen. First record for this species from KSA.

**Remarks.** The species was collected by SW on *Ind. oblongifolia* and *Ael. lagopoides* in limestone areas and from sand dunes, respectively. Identification by A.N. Al Ansi.

**87. *Scymnus subvillosus* (Goeze, 1777)**

**Material examined. Sajid Island**: Sajid, 16°45'42.6"N, 41°59'56.0"E, 6 m, 27.i.2017, 1 (SW); 5.iii.2017, 1 (SW).

**Literature records.** Asir and Riyadh (Abdel-Dayem et al. 2017).

**General distribution.** AFR_PAL_SAR species, widely distributed through Western to Eastern Europe and Central Asia, southward to Southern Europe, North Africa, Levant, Arabian Peninsula (KSA, Kuwait, Qatar, United Arab Emirates, Yemen), and Iran to Pakistan.

**Remarks.** The specimens were collected by SW on *Sen. alexandrina* from sand dunes. Identification by A.N. Al Ansi.

**88. *Scymnus syriacus* (Marsuel, 1868)**

**Material examined. Sajid Island**: Khotp, 16°52'27.9"N, 41°54'43.9"E, 9 m, 27.i.2017, 1 (SW). Khotp, 16°52'47.6"N, 41°54'39.7"E, 11 m, 5.iii.2017, 1 (SW).

**Literature records.** Baha, Eastern Province, Riyadh (Abdel-Dayem et al. 2017).

**General distribution.**SAR species, distributed in Egypt, east to Levant, Arabian Peninsula (KSA), and Iran.

**Remarks.** The species was collected by SW on *Ind. oblongifolia* in limestone areas. Identification by A.N. Al Ansi.

**89. *Scymnus yemenensis* (Kapur, 1959)**

**Material examined. Sajid Island**: Khotp, 16°52'27.9"N, 41°54'43.9"E, 9 m, 18.ii.2017, 1 (SW).

**Literature records.** Asir and Riyadh (Abdel-Dayem et al. 2017).

**General distribution.** END_AR species, an endemic of the southern Arabian Peninsula (KSA, Oman, United Arab Emirates, Yemen).

**Remarks.** The unique listed specimen was collected by SW on *Ind. oblongifolia* in a limestone area. Identification by A.N. Al Ansi.

#### Family Laemophloeidae

**90. *Placonotus testaceus* (Fabricius, 1787)**

**Material examined. Sajid Island**: Sajid, 16°45'42.6"N, 41°59'56.0"E, 6 m, 10.vi.2017, 1 (LT); 13.x.2017, 1 (LT).

**Literature records.** Riyadh (Abdel-Dayem et al. 2017).

**General distribution.**SCO species, cited from North Africa, Arabian Peninsula (KSA), north to eastern, northern, southern, and western Europe and Central Asia; from India east to Japan, south to Australia.

**Remarks.** The species was collected by LT in sand dunes. Identification by H.H. Fadl.

#### Family Phalacridae

**91. *Phalacrus substriatus* Gyllenhal, 1813**

**Material examined. Sajid Island**: Sajid, 16°45'42.6"N, 41°59'56.0"E, 6 m, 28.i.2017, 3 (SW).

**General distribution.**PAL species, widely distributed in eastern, northern, and southern Europe. New record for KSA.

**Remarks.** The listed specimens were collected by SW on *Sen. alexandrina* in sand dunes. Identification by H.H. Fadl.

#### Family Silvanidae

**92. *Oryzaephilus surinamensis* (Linnaeus, 1758)**

**Material examined. Sajid Island**: Bridge, 16°51'27.4"N, 41°55'49.0"E, 2 m, 10.xi.2017, 2 (SW). Khotp, 16°52'27.9"N, 41°54'43.9"E, 9 m, 25.i.2017, 2 (SW); 2.xii.2017, 1 (SW). Khotp, 16°52'47.6"N, 41°54'39.7"E, 11 m, 10.xi.2017, 1 (SW). Sajid, 16°45'42.6"N, 41°59'56.0"E, 6 m, 27.i.2017, 1 (SW); 5.iii.2017, 3 (LT); 10.vi.2017, 3 (LT); 13.x.2017, 32 (LT); 10.xi.2017, 3 (LT); 3.xii.2017, 3 (LT).

**Literature records.** Baha and Makkah (Ślipiński 1984; El-Hawagry et al. 2013).

**General distribution.**COS species, cosmopolitan.

**Remarks.** The specimens of this species were collected by SW on *Ind. oblongifolia* and by LT in limestone areas; and SW on *Aer. javanica*, *Abu. pannosum*, and *Ind. oblongifolia* in sand dunes. Identification by H.H. Fadl.

#### Superfamily Curculionoidea

**Family Brentidae**

**93. *Aspidapion* sp.**

**Material examined. Sajid Island**: Sajid, 16°45'42.6"N, 41°59'56.0"E, 6 m, 5.iii.2017, 1 (SW).

**Remarks.** The listed single specimen was collected by SW in a sand dune.

**94. *Perapion* sp.**

**Material examined. Farasan Island**: Al-Huseis, 16°45'20.8"N, 42°03'59.6"E, 7 m, 5.iii.2017, 5 (SW); 2.iv.2017, 3 (SW); 9.vi.2017, 2 (SW); 28.vii.2017, 3 (SW); 5.ix.2017, 3 (SW). Al-Kosar, 16°40'14.8"N, 42°08'54.9"E, 7 m, 2.iv.2017, 5 (SW); 9.vi.2017, 3 (SW); 5.ix.2017, 4 (SW). Farasan Reserve, 16°42'26.5"N, 42°04'19.2"E, 7 m, 9.vi.2017, 1 (SW). **Sajid Island**: Bridge, 16°51'27.4"N, 41°55'49.0"E, 2 m, 27.i.2017, 1 (SW); 9.vi.2017, 2 (SW); 5.ix.2017, 4 (SW); 10.xi.2017, 11 (SW). Khotp, 16°52'27.9"N, 41°54'43.9"E, 9 m, 27.i.2017, 4 (SW); 18.ii.2017, 16 (SW); 5.iii.2017, 33 (SW); 23.v.2017, 2 (SW); 9.vi.2017, 24 (SW); 5.ix.2017, 20 (SW); 10.xi.2017, 3 (SW); 2.xii.2017, 11 (SW). Khotp, 16°52'47.6"N, 41°54'39.7"E, 11 m, 27.i.2017, 2 (SW); 17.ii.2017, 3 (SW); 5.iii.2017, 21 (SW); 5.iii.2017, 14 (SW); 23.v.2017, 8 (SW); 9.vi.2017, 4 (SW); 5.ix.2017, 36 (SW); 10.xi.2017, 2 (SW); 2.xii.2017, 5 (SW). Sajid, 16°45'42.6"N, 41°59'56.0"E, 6 m, 26.i.2017, 2 (LT); 27.i.2017, 4 (SW); 5.iii.2017, 4 (SW); 9.vi.2017, 5 (SW); 5.ix.2017, 15 (SW); 10.xi.2017, 3 (SW).

**Remarks.** This unidentified species was collected by SW on *Sal. persica* in coastal sandy areas, *Ind. oblongifolia* in limestone areas, *Sua. monoica* in salt marshes, and on *Aer. javanica*, *Ind. oblongifolia*, *Lim. axillare*, and *Sen. alexandrina* in sand dunes.

#### Family Curculionidae

**95. *Acythopeus curvirostris granulipennis* (Tournier, 1873)**

**Material examined. Sajid Island**: Khotp, 16°52'47.6"N, 41°54'39.7"E, 11 m, 5.ix.2017, 1 (SW). Sajid, 16°45'42.6"N, 41°59'56.0"E, 6 m, 28.i.2017, 1 (SW).

**Literature records.** Hail, Jizan, Jouf, Madinah, Makkah, Riyadh, and Tabouk (Abu-Thuraya 1982).

**General distribution.** AFR_ORR_SAR species, distributed in Sahel, northward to North Africa (Algeria, Egypt), east across Levant, Arabian Peninsula (KSA, Oman, United Arab Emirates), Iran to India.

**Remarks.** The species was collected by SW on *Ind. oblongifolia* and *Sen. alexandrina* in limestone areas and from sand dunes, respectively. Identification by E. Colonnelli.

**96. *Amblyrhinus cylindricollis* Magnano, 2009**

**Material examined. Farasan Island**: Al-Huseis, 16°45'20.8"N, 42°03'59.6"E, 7 m, 17.ii.2017, 1 (SW). Al-Kosar, 16°40'14.8"N, 42°08'54.9"E, 7 m, 17.ii.2017, 1 (SW). Farasan Reserve, 16°42'26.5"N, 42°04'19.2"E, 7 m, 17.ii.2017, 8 (SW). **Sajid Island**: Bridge, 16°51'27.4"N, 41°55'49.0"E, 2 m, 25.i.2017, 1 (SW); 18.ii.2017, 3 (SW); 9.vi.2017, 3 (SW). Khotp, 16°52'27.9"N, 41°54'43.9"E, 9 m, 18.ii.2017, 3 (SW). Khotp, 16°52'47.6"N, 41°54'39.7"E, 11 m, 18.ii.2017, 1 (SW). Farasan Reserve, 16°42'26.3"N, 42°03'59.4"E, 5 m, 17.ii.2017, 2 (SW). Sajid, 16°45'42.6"N, 41°59'56.0"E, 6 m, 25.i.2017, 1 (LT); 18.ii.2017, 2 (SW); 5.iii.2017, 2 (SW).

**General distribution.** END_AR species, recently was described from United Arab Emirates (Magnano et al. 2009). New country record for KSA.

**Remarks.** This species was collected by SW on *Sal. persica* in coastal sandy areas, *Ind. oblongifolia* in limestone areas, *Sua. monoica* in salt marshes, and *Aer. javanica*, *Ind. oblongifolia*, *Lim. axillare*, and *Sen. alexandrina* in sand dunes. Identification by E. Colonnelli.

**97. *Cosmogaster cordofanus* (Fåhraeus, 1842)**

**Material examined. Sajid Island**: Sajid, 16°45'42.6"N, 41°59'56.0"E, 6 m, 10.xi.2017, 1 (LT); 3.xii.2017, 1 (LT).

**Literature records.** Previous specific locality information for KSA records was not available to us.

**General distribution.** AFR_SAR species, distributed through Sahel, north to Egypt, east to Arabian Peninsula (KSA, United Arab Emirates).

**Remarks.** The specimens were collected by LT in sand dunes. Identification by E. Colonnelli.

**98. *Dereodus phasianellus* (Fairmaire, 1886)**

**Material examined. Farasan Island**: Al-Huseis, 16°45'20.8"N, 42°03'59.6"E, 7 m, 26.i.2017, 1 (SW). Al-Kosar, 16°40'14.8"N, 42°08'54.9"E, 7 m, 10.x.2017, 1 (SW). Farasan Reserve, 16°42'26.5"N, 42°04'19.2"E, 7 m, 10.x.2017, 1 (SW). **Sajid Island**: Bridge, 16°51'27.4"N, 41°55'49.0"E, 2 m, 27.iv.2017, 1 (SW). Khotp, 16°52'27.9"N, 41°54'43.9"E, 9 m, 10.x.2017, 1 (SW); 10.x.2017, 1 (SW). Khotp, 16°52'47.6"N, 41°54'39.7"E, 11 m, 25.i.2017, 11 (SW); 25.i.2017, 11 (SW); 5.iii.2017, 1 (SW). Sajid, 16°45'42.6"N, 41°59'56.0"E, 6 m, 25.i.2017, 1 (SW); 17.ii.2017, 2 (LT).

**General distribution.**AFR species, distributed in the Horn of Africa, east to Arabian Peninsula (Yemen). New country record for KSA.

**Remarks.** This species was collected by SW on *Sal. persica* in coastal sandy areas, *Ind. oblongifolia* in limestone areas, *Sua. monoica* in salt marshes, and *Lim. axillare* and *Sen. alexandrina* in sand dunes. Identification by E. Colonnelli.

**99. *Dereodus* sp.**

**Material examined. Sajid Island**: Khotp, 16°52'27.9"N, 41°54'43.9"E, 9 m, 2.xii.2017, 1 (SW).

**Remarks.** The unique listed specimen was collected by SW on *Ind. oblongifolia* in a limestone area.

**100. *Mecinus paratychioides* (Hoffmann, 1965)**

**Material examined. Sajid Island**: Khotp, 16°52'47.6"N, 41°54'39.7"E, 11 m, 25.i.2017, 1 (SW). Sajid, 16°45'42.6"N, 41°59'56.0"E, 6 m, 2.xii.2017, 1 (SW); 5.iii.2017, 3 (SW).

**Literature records.** Riyadh (Caldara and Fogato 2013).

**General distribution.**SAR species, distributed in Western Sahara from Morocco to Libya, east to Arabian Peninsula (KSA, United Arab Emirates).

**Remarks.** The species was collected by SW on *Ind. oblongifolia* and *Sen. alexandrina* in limestone areas and from sand dunes, respectively. Identification by E. Colonnelli.

**101. *Myllocerus* sp.**

**Material examined. Farasan Island**: Al-Huseis, 16°45'20.8"N, 42°03'59.6"E, 7 m, 17.ii.2017, 1 (SW). Farasan Reserve, 16°42'26.3"N, 42°03'59.4"E, 5 m, 17.ii.2017, 1 (SW). **Sajid Island**: Bridge, 16°51'27.4"N, 41°55'49.0"E, 2 m, 18.ii.2017, 1 (SW). Khotp, 16°52'27.9"N, 41°54'43.9"E, 9 m, 18.ii.2017, 1 (SW); 5.iii.2017, 4 (SW); 10.xi.2017, 1 (SW). Khotp, 16°52'47.6"N, 41°54'39.7"E, 11 m, 17.ii.2017, 14 (SW); 5.iii.2017, 11 (SW); 10.xi.2017, 1 (SW). Sajid, 16°45'42.6"N, 41°59'56.0"E, 6 m, 25.i.2017, 4 (SW); 25.i.2017, 2 (LT); 17.ii.2017, 2 (LT); 18.ii.2017, 1 (SW); 27.iv.2017, 5 (LT); 10.xi.2017, 2 (SW); 3.xii.2017, 2 (LT).

**Remarks.** The listed specimens were collected by SW on *Sal. persica* in coastal sandy areas, *Ind. oblongifolia* in limestone areas, *Sua. monoica* in salt marshes; and *Lim. axillare* and *Sen. alexandrina* in sand dunes.

**102. *Sharpia sabulicola* Colonnelli, 2009**

**Material examined. Sajid Island**: Khotp, 16°52'27.9"N, 41°54'43.9"E, 9 m, 5.ix.2017, 1 (SW).

**Literature records.** Riyadh (Abdel-Dayem et al. 2017).

**General distribution.** END_AR species, previously recorded from Arabian Peninsula (KSA United Arab).

**Remarks.** This record is the second locality documented for KSA. The single listed specimen was collected by SW in a limestone area. Identification by E. Colonnelli.

**103. *Tychius vicinus* Roudier, 1954**

**Material examined. Farasan Island**: Al-Kosar, 16°40'14.8"N, 42°08'54.9"E, 7 m, 25.vi.2017, 2 (SW). **Sajid Island**: Sajid, 16°45'42.6"N, 41°59'56.0"E, 6 m, 18.ii.2017, 1 (SW).

**Literature records.** Asir and Riyadh (Abdel-Dayem et al. 2017).

**General distribution.**SAR species, distributed in North Africa (Algeria, Egypt), east to Arabian Peninsula (KSA, United Arab Emirates, Yemen).

**Remarks.** The species was collected by SW on *Lim. axillare* and *Sen. alexandrina* in sand dunes. Identification by E. Colonnelli.

#### Superfamily Elateroidea

**Family Elateridae**

**104. *Aeoloides grisescens* (Germar, 1844)**

**Material examined. Farasan Island**: Al-Huseis, 16°45'20.8"N, 42°03'59.6"E, 7 m, 17.ii.2017, 1 (PT); 3.xii.2017, 1 (PT). Al-Kosar, 16°40'14.8"N, 42°08'54.9"E, 7 m, 17.ii.2017, 1 (PT); 13.x.2017, 1 (SW). Farasan Reserve, 16°42'26.5"N, 42°04'19.2"E, 7 m, 13.x.2017, 1 (SW). **Sajid Island**: Bridge, 16°51'27.4"N, 41°55'49.0"E, 2 m, 18.ii.2017, 2 (PT); 3.iii.2017, 3 (PT); 23.v.2017, 2 (SW). Khotp, 16°52'27.9"N, 41°54'43.9"E, 9 m, 13.x.2017, 2 (SW). Khotp, 16°52'47.6"N, 41°54'39.7"E, 11 m, 18.ii.2017, 1 (PT); 13.x.2017, 2 (SW). Sajid, 16°45'42.6"N, 41°59'56.0"E, 6 m, 25.i.2017, 4 (SW); 25.i.2017, 1 (PT); 18.ii.2017, 2 (PT); 5.iii.2017, 3 (LT); 5.iii.2017, 2 (PT); 28.iv.2017, 2 (LT); 28.vii.2017, 1 (LT); 6.ix.2017, 1 (LT); 13.x.2017, 2 (LT); 13.x.2017, 1 (SW); 10.xi.2017, 4 (LT).

**Literature records.** Eastern Province, Jizan, Qaseem, Riyadh (Abdel-Dayem et al. 2017).

**General distribution.** PAL_SAR species, widely distributed in the Sahara Desert through North Africa east to Levant, Arabian Peninsula (KSA, Oman, Qatar, United Arab Emirates, Yemen), Iran, Afghanistan, Pakistan; north to Greece, Ukraine, Central Asia.

**Remarks.** This species was collected by PT and SW in coastal sandy areas, limestone areas, salt marshes, and sand dunes. Identification by H.M. Aldhafer.

**105. *Agrypnus omanensis* Platia & Schimmel, 1997**

**Material examined. Sajid Island**: Khotp, 16°52'27.9"N, 41°54'43.9"E, 9 m, 10.x.2017, 1 (SW). Khotp, 16°52'47.6"N, 41°54'39.7"E, 11 m, 10.x.2017, 2 (SW). Sajid, 16°45'42.6"N, 41°59'56.0"E, 6 m, 5.iii.2017, 1 (LT); 23.v.2017, 1 (LT); 10.xi.2017, 1 (LT).

**General distribution.** END_AR species, until now cited from Oman. New country record for KSA.

**Remarks.** The listed specimens were collected by SW on *Ind. oblongifolia* in limestone areas and by LT in sand dunes. Identification by H.M. Aldhafer.

**106. *Cardiophorus niger* Platia, 2012**

**Material examined. Sajid Island**: Sajid, 16°45'42.6"N, 41°59'56.0"E, 6 m, 13.x.2017, 1 (LT).

**General distribution.** END_AR species, until now reported from Yemen. New country record for KSA.

**Remarks.** The listed unique specimen was collected by LT in a sand dune. Identification by H.M. Aldhafer.

**107. *Craspedostethus dilutus* (Erichson 1840)**

**Material examined. Sajid Island**: Sajid, 16°45'42.6"N, 41°59'56.0"E, 6 m, 25.i.2017, 1 (SW); 5.iii.2017, 2 (LT); 28.iv.2017, 5 (LT); 23.v.2017, 2 (LT); 10.vi.2017, 3 (LT); 12.viii.2017, 8 (LT); 13.x.2017, 8 (LT); 10.xi.2017, 5 (LT); 3.xii.2017, 10 (LT).

**Literature records.** Baha, Eastern Province, Makkah, and Riyadh (Abdel-Dayem et al. 2017).

**General distribution.** AFR_SAR species, distributed in the Horn of Africa, north to North Africa, east to Arabian Peninsula (KSA, Oman, United Arab Emirates, Yemen), Syria, Iran.

**Remarks.** The listed specimens were frequently found in sand dunes and collected by SW on *Sen. alexandrina* and by LT. Identification by H.M. Aldhafer.

**108. *Dicronychus bicoloratus* Platia, 2012**

**Material examined. Sajid Island**: Sajid, 16°45'42.6"N, 41°59'56.0"E, 6 m, 13.x.2017, 1 (LT); 10.xi.2017, 1 (LT).

**General distribution.** END_AR species, only known from Yemen. New country record for KSA.

**Remarks.** The species was collected by LT in sand dunes. Identification by H.M. Aldhafer.

**109. *Drasterius aegyptiacus* Buysson 1905**

**Material examined. Sajid Island**: Sajid, 16°45'42.6"N, 41°59'56.0"E, 6 m, 10.vi.2017, 4 (LT); 3.xii.2017, 8 (LT).

**Literature records.** Asir, Eastern Province, Hail, Jizan, Makkah and Riyadh (Chassain 1979, 1983; Platia and Schimmel 1997).

**General distribution.**SAR species, distributed through entire the North Africa, east to Arabian Peninsula (KSA, Yemen).

**Remarks.** This species was redescribed by Chassain (1979) as *Drasterius lateralis riyadhensis* from Riyadh Province, but subsequently Platia and Schimmel (1997) synonymized it with *D. aegyptiacus*. Our specimens were collected by LT in sand dunes. Identification by H.M. Aldhafer.

**110. *Lanelater wittmeri* Chassain, 1983**

**Material examined. Sajid Island**: Sajid, 16°45'42.6"N, 41°59'56.0"E, 6 m, 10.vi.2017, 4 (LT); 6.ix.2017, 1 (LT).

**Literature records.** This species was previously known only from its type locality in Asir Province (Chassain 1983).

**General distribution.** END_SA species, known only from KSA.

**Remarks.** This is the first report of this species since it was first described in Asir (Chassain 1983). The listed specimens were collected by LT in sand dunes. Identification by H.M. Aldhafer.

**111. *Meristhus lepidotus* (Palisot de Beauvois, 1805)**

**Material examined. Sajid Island**: Sajid, 16°45'42.6"N, 41°59'56.0"E, 6 m, 26.i.2017, 1 (LT); 6.ix.2017, 1 (LT); 13.x.2017, 1 (LT); 10.xi.2017, 1 (LT).

**Literature records.** Makkah (Platia and Schimmel 1997).

**General distribution.**AFR species, recorded from Chad, Somalia, Arabian Peninsula (KSA).

**Remarks.** The specimens of this species were collected by LT in sand dunes. Identification by H.M. Aldhafer.

**112. *Selasia arabica* Geisthardt, 2003**

**Material examined. Sajid Island**: Sajid, 16°45'42.6"N, 41°59'56.0"E, 6 m, 5.iii.2017, 1 (LT).

**Literature records.** Jizan (Trllova and Kundrata 2015)

**General distribution.** END_AR species, reported from Arabian Peninsula (KSA, Oman, Yemen).

**Remarks.***Selasia arabica* is only known, in KSA, from a single specimen (without head and prothorax) collected in 1982 from Fifa (Jizan Province) (Trllova and Kundrata 2015). This second specimen was collected by LT in a sand dune. Identification by H.M. Aldhafer.

#### Superfamily Histeroidea

**Family Histeridae**

**113. *Saprinus chalcites* (Illiger, 1807)**

**Material examined. Farasan Island**: Farasan Reserve, 16°42'26.5"N, 42°04'19.2"E, 7 m, 5.iii.2017, 2 (PT).

**Literature records.** Asir, Baha, Jizan, Makkah, Najran, Riyadh (Abdel-Dayem et al. 2017).

**General distribution.**SCO species (Penati and Vienna 2006), widely distributed through the entire Sahar Desert to southeast Asia, south to Africa and Australia, north to southern Europe and Central Asia, also reported from South America.

**Remarks.** This species was collected by PT under the canopy of *Zyg. coccineum* in salt marshes. Identification by M.S. Abdel-Dayem.

**114. *Saprinus gilvicornis* Erichson, 1834**

**Material examined. Sajid Island**: Khotp, 16°52'27.9"N, 41°54'43.9"E, 9 m, 5.iii.2017, 1 (PT).

**Literature records.**Dahlgren (1974) mentioned KSA among the distribution of this species without providing an exact locality.

**General distribution.** PAL_SAR species, cited from Chad, Sudan, Egypt, Arabian Peninsula (KSA, Oman), and Central Asia (Kazakhstan, Turkmenistan).

**Remarks.** The unique listed specimen was collected by PT under the canopy of in a limestone area. Identification by M.S. Abdel-Dayem.

**115. *Saprinus moyses* Marseul, 1862**

**Material examined. Farasan Island**: Al-Kosar, 16°40'14.8"N, 42°08'54.9"E, 7 m, 4.iii.2017, 1 (PT). Farasan Reserve, 16°42'26.3"N, 42°03'59.4"E, 5 m, 17.ii.2017, 2 (PT).

**Literature records.** Hail and Riyadh (Abdel-Dayem et al. 2017).

**General distribution.**SAR species, distributed through North Africa, from Morocco east to Levant, Arabian Peninsula (KSA, Kuwait); reaching its northern range in Spain.

**Remarks.** The species was collected by PT under the canopies of *Ble. edulis* and *Lim. axillare* in salt marches and sand dunes, respectively. Identification by M.S. Abdel-Dayem.

#### Superfamily Hydrophiloidea

**Family Hydrophilidae**

**116. *Berosus nigriceps* (Fabricius, 1801)**

**Material examined. Sajid Island**: Sajid, 16°45'42.6"N, 41°59'56.0"E, 6 m, 13.x.2017, 1 (LT).

**Literature records.** Eastern Province, Jizan (Hebauer 1997)

**General distribution.** AFR_ORR_SAR species, distributed in southern Africa, Sahel, east from Arabian Peninsula (KSA, Oman, United Arab Emirates, Yemen), Iraq and Iran to India, Nepal, and Bangladesh.

**Remarks.** This single listed specimen was collected by LT near a temporary freshwater pool in sandy area. Identification by M.S. Abdel-Dayem.

**117. *Berosus rubiginosus* Kuwert, 1890**

**Material examined. Sajid Island**: Sajid, 16°45'42.6"N, 41°59'56.0"E, 6 m, 10.xi.2017, 2 (LT).

**Literature records.** Asir, Baha, Jizan, Madinah, Makkah, Najran, Riyadh and Tabouk (Hebauer 1997).

**General distribution.**AFR species, widely distributed in Africa and cited from southern Arabian Peninsula (KSA, Oman, United Arab Emirates, Yemen).

**Remarks.** This species was collected by LT near a temporary freshwater pool in sandy area. Identification by M.S. Abdel-Dayem.

#### Superfamily Scarabaeoidea

**Family Glaresidae**

**118. *Glaresis arabica* (Paulian, 1980)**

**Material examined. Sajid Island**: Sajid, 16°45'42.6"N, 41°59'56.0"E, 6 m, 2.xii.2017, 1 (LT).

**Literature records.** Eastern Province and Riyadh (Abdel-Dayem et al. 2017).

**General distribution.** END_AR species, only known from Oman and KSA.

**Remarks.** The unique found specimen was collected by LT in a sand dune. Identification by D. Král.

#### Family Hybosoridae

**119. *Hybosorus illigeri* Reiche, 1853**

**Material examined. Sajid Island**: Sajid, 16°45'42.6"N, 41°59'56.0"E, 6 m, 17.ii.2017, 2 (LT); 5.iii.2017, 2 (LT); 28.iv.2017, 2 (LT); 10.vi.2017, 3 (LT); 28.vii.2017, 6 (LT); 12.viii.2017, 3 (LT); 6.ix.2017, 4 (LT); 13.x.2017, 4 (LT); 10.xi.2017, 14 (LT); 2.xii.2017, 4 (LT).

**Literature records.** Eastern Province, Madinah, Makkah, Najran, and Riyadh (Abdel-Dayem et al. 2017).

**General distribution.**COS species, cosmopolitan.

**Remarks.** This species was frequently collected by LT in sand dunes. Identification by D. Král.

#### Family Scarabaeidae

**120. *Ablaberoides abyssinicus* (Brenske 1902)**

**Material examined. Sajid Island**: Khotp, 16°52'27.9"N, 41°54'43.9"E, 9 m, 25.i.2017, 2 (SW). Khotp, 16°52'47.6"N, 41°54'39.7"E, 11 m, 25.i.2017, 1 (SW). Sajid, 16°45'42.6"N, 41°59'56.0"E, 6 m, 25.i.2017, 2 (LT); 25.i.2017, 6 (SW); 17.ii.2017, 25 (LT); 5.iii.2017, 2 (LT); 10.iv.2017, 3 (LT); 12.viii.2017, 10 (LT); 6.ix.2017, 3 (LT); 10.xi.2017, 8 (LT); 2.xii.2017, 5 (LT).

**Literature records.** Asir, Eastern Province, Jizan, Madinah, Makkah, and Riyadh (Ahrens 2000).

**General distribution.**AFR species, distributed in Horn of Africa (Ethiopia, Somalia), Chad, North Africa (Egypt, Morocco), east to Arabian Peninsula (KSA, Oman, United Arab Emirates, Yemen).

**Remarks.** The listed specimens were collected by SW on *Ind. oblongifolia* and by LT in limestone areas and sand dunes. Identification by D. Král.

**121. *Adoretus* sp.**

**Material examined. Sajid Island**: Sajid, 16°45'42.6"N, 41°59'56.0"E, 6 m, 2.xii.2017, 1 (LT); 10.xi.2017, 1 (LT).

**Remarks.** This unidentified species was collected by LT in sand dunes.

**122. *Aphodius adustus* Klug, 1855**

**Material examined. Sajid Island**: Khotp, 16°52'47.6"N, 41°54'39.7"E, 11 m, 2.xii.2017, 1 (SW). Sajid, 16°45'42.6"N, 41°59'56.0"E, 6 m, 13.x.2017, 3 (LT); 2.xii.2017, 1 (LT).

**Literature records.** Riyadh (Abdel-Dayem et al. 2017).

**General distribution.**AFR species, widely distributed in the Sub-Saharan Africa, east to southwestern Arabian Peninsula (KSA, Yemen).

**Remarks.** This species was collected by SW on *Ind. oblongifolia* in limestone area and by LT in sand dunes. Identification by D. Král.

**123. *Eremazus unistriatus* Mulsant, 1851**

**Material examined. Sajid Island**: Sajid, 16°45'42.6"N, 41°59'56.0"E, 6 m, 10.iv.2017, 7 (LT); 13.x.2017, 6 (LT); 10.xi.2017, 1 (LT).

**Literature records.** Eastern Province, Hail, Madinah, Makkah, Qaseem, and Riyadh (Abdel-Dayem et al. 2017).

**General distribution.** ORR_PAL_SAR species, widely distributed in the Sahara Desert, from Morocco east through Levant and Arabian Peninsula (KSA, United Arab Emirates) to Pakistan; northwards to Spain, south Caucasus, and Central Asia.

**Remarks.** The listed specimens were collected by LT in sand dunes. Identification by D. Král.

**124. *Granulopsammodius plicatulus* (Fairmaire, 1892)**

**Material examined. Sajid Island**: Bridge, 16°51'27.4"N, 41°55'49.0"E, 2 m, 10.iv.2017, 1 (SW). Sajid, 16°45'42.6"N, 41°59'56.0"E, 6 m, 17.ii.2017, 1 (LT); 10.iv.2017, 4 (LT); 6.vii.2017, 2 (LT); 12.viii.2017, 3 (LT); 6.ix.2017, 2 (LT); 13.x.2017, 6 (LT); 3.xii.2017, 5 (LT).

**Literature records.** Asir, Baha, Eastern Province, Madinah, Makkah, and Riyadh (Abdel-Dayem et al. 2017).

**General distribution.** AFR_SAR species, distributed in East Africa, north to North Africa, east to Arabian Peninsula (KSA, Yemen (Socotra)).

**Remarks.** This species was frequently collected in sand dunes by SW on *Dic. foveolatum* and *Ind. oblongifolia* and by LT. Identification by D. Král.

**125. *Gymnopleurus* sp.**

**Material examined. Sajid Island**: Khotp, 16°52'27.9"N, 41°54'43.9"E, 9 m, 6.vii.2017, 2 (PT).

**Remarks.** This unidentified species was collected by PT under *Ind. spinosa* in a limestone area.

**126. *Homothyrea thoracica* (Schaum, 1841)**

**Material examined. Sajid Island**: Sajid, 16°45'42.6"N, 41°59'56.0"E, 6 m, 25.i.2017, 2 (SW); 23.v.2017, 1 (SW).

**General distribution.**AFR species, distributed in the Horn of Africa (Ethiopia, Somalia) and the southwestern Arabian Peninsula (Yemen). New country record for KSA.

**Remarks.** This species was collected by SW vegetation in sand dunes. Identification by D. Král.

**127. *Onthophagus sticticus* Harold, 1867**

**Material examined. Sajid Island**: Sajid, 16°45'42.6"N, 41°59'56.0"E, 6 m, 17.ii.2017, 1 (LT); 10.xi.2017, 5 (LT); 3.xii.2017, 1 (LT).

**Literature records.** Jizan, Madinah, and Makkah (Ziani et al. 2019).

**General distribution.** AFR_SAR species, distributed East and North Africa, Arabian Peninsula (KSA, Oman, United Arab Emirates, Yemen), and Iran.

**Remarks.** The listed specimens were collected by LT in sand dunes. Identification by S. Ziani.

**128. *Onthophagus variegatus* (Fabricius, 1798)**

**Material examined. Sajid Island**: Khotp, 16°52'27.9"N, 41°54'43.9"E, 9 m, 6.vii.2017, 1 (LT). Sajid, 16°45'42.6"N, 41°59'56.0"E, 6 m, 23.v.2017, 1 (LT); 10.xi.2017, 3 (LT); 2.xii.2017, 2 (LT).

**Literature records.** Asir, Baha, Jizan, Makkah, and Riyadh (Ziani et al. 2019).

**General distribution.** AFR_SAR species, widely distributed in the Sub-Saharan Africa, Egypt, east from Arabian Peninsula (KSA, Oman, United Arab Emirates, Yemen) and Iraq to Pakistan.

**Remarks.** This species was frequently found in sand dunes and collected by LT. Identification by S. Ziani.

**129. *Pentodon algerinus dispar* Baudi, 1870**

**Material examined. Sajid Island**: Sajid, 16°45'42.6"N, 41°59'56.0"E, 6 m, 23.v.2017, 1 (LT); 6.ix.2017, 2 (LT); 3.xii.2017, 2 (LT).

**Literature records.** Eastern Province, Makkah, and Riyadh, KSA (Abdel-Dayem et al. 2017).

**General distribution.**SAR species, distributed through the Sahara Desert, in Levant, Arabian Peninsula (KSA, Kuwait, Oman, Qatar, Yemen), Iran, but extended its range to Southern Europe (Greece) and Africa (Eretria).

**Remarks.** The above-listed specimens were collected by LT in sand dunes. Identification by D. Král.

**130. *Rhyssemus granosus* (Klug & Erichson, 1842)**

**Material examined. Sajid Island**: Sajid, 16°45'42.6"N, 41°59'56.0"E, 6 m, 25.i.2017, 4 (LT); 17.ii.2017, 2 (LT); 5.iii.2017, 3 (LT); 6.ix.2017, 4 (LT); 10.xi.2017, 1 (LT); 2.xii.2017, 1 (LT).

**Literature records.** Asir, Baha, Eastern Province, Jizan, Madinah, Makkah, Qaseem, and Riyadh (Abdel-Dayem et al. 2017).

**General distribution.**AFR species, distributed in Sub-Saharan Africa, from Senegal eastward to East Africa and Arabian Peninsula (KSA, Yemen (Socotra)); also cited from Egypt.

**Remarks.** The species was frequently found in sand dunes and collected by LT. Identification by D. Král.

**131. *Rhyssemus saoudi* Pittino, 1984**

**Material examined. Sajid Island**: Sajid, 16°45'42.6"N, 41°59'56.0"E, 6 m, 25.i.2017, 5 (LT); 17.ii.2017, 3 (LT); 5.iii.2017, 2 (LT); 10.iv.2017, 2 (LT); 10.vi.2017, 34 (LT); 6.ix.2017, 3 (LT); 13.x.2017, 2 (LT); 10.xi.2017, 1 (LT).

**Literature records.** Asir, Baha, Jizan, Makkah, and Riyadh (Abdel-Dayem et al. 2017).

**General distribution.** END_SA species, only known from KSA.

**Remarks.** These specimens were frequently collected in sand dunes by LT. Identification by D. Král.

**132. *Scarabaeus krutai* Maly & Montreuil, 2011**

**Material examined. Sajid Island**: Khotp, 16°52'47.6"N, 41°54'39.7"E, 11 m, 18.ii.2017, 1 (PT). Khotp, 16°52'47.6"N, 41°54'39.7"E, 11 m, 5.iii.2017, 1 (PT).

**Literature records.** The species is known only from its type locality in the southwestern high mountains of Asir (Kamis Mushayt) and Hijaz (Makkah, Taif) (Maly and Montreuil 2011).

**General distribution.** END_SA species, only known from KSA.

**Remarks.** The specimens were collected from much lower altitude and are considered the second record of *S. krutai* from southwestern KSA. The species was collected by PT under the canopy of *Ind. oblongifolia* in limestone areas. Identification by D. Král.

#### Family Trogidae

**133. *Omorgus niloticus desertorum* (Harold, 1872)**

**Material examined. Farasan Island**: Farasan Reserve, 16°42'26.3"N, 42°03'59.4"E, 5 m, 5.iii.2017, 1 (PT). Farasan Reserve, 16°42'26.5"N, 42°04'19.2"E, 7 m, 5.iv.2017, 1 (PT). **Sajid Island**: Khotp, 16°52'27.9"N, 41°54'43.9"E, 9 m, 13.x.2017, 1 (PT).

**Literature records.** This species has been documented from Asir, Jizan, and Makkah provinces of KSA (Scholtz 1980), but was not listed from KSA in the Catalogue of Palaearctic Coleoptera (Pittino 2006).

**General distribution.** AFR_MAD species, distributed in East Africa (Eretria, Ethiopia), Madagascar, Egypt, and Arabian Peninsula (KSA, United Arab Emirates, Yemen).

**Remarks.** This species was collected by PT under the canopies of *Ind. oblongifolia* and *Ble. edulis* in limestone areas and salt marches, respectively. Identification by H.H. Fadl.

**134. *Omorgus verrucosus* (Reiche & Saulcy, 1856)**

**Material examined. Sajid Island**: Khotp, 16°52'27.9"N, 41°54'43.9"E, 9 m, 5.iii.2017, 1 (PT); 18.ix.2017, 1 (PT). Khotp, 16°52'47.6"N, 41°54'39.7"E, 11 m, 18.ix.2017, 1 (PT). Sajid, 16°45'42.6"N, 41°59'56.0"E, 6 m, 13.x.2017, 1 (LT).

**General distribution.**SAR species, cited from Egypt, Levant, and Arabian Peninsula (United Arab Emirates, Yemen). New country record for KSA.

**Remarks.** The listed specimens were collected by PT under the canopy of *Ind. oblongifolia* in limestone areas and by LT in sand dune. Identification by H.H. Fadl.

#### Superfamily Staphylinoidea

**Family Staphylinidae**

**135. *Bledius capra* Fauvel, 1875**

**Material examined. Sajid Island**: Sajid, 16°45'42.6"N, 41°59'56.0"E, 6 m, 10.vi.2017, 1 (LT); 10.xi.2017, 5 (LT).

**Literature records.** Tabouk (Gusarov 1997).

**General distribution.** AFR_SAR species, distributed in East and North Africa, east to Arabian Peninsula (KSA, Oman, Yemen), Iran; reaches it northern limits at Turkey.

**Remarks.** This species is confined to the Red Sea coastal regions of KSA (Gusarov 1997). The above-listed specimens were collected by LT in sand dunes. Identification by A.A. Elgharbawy.

**136. *Bledius niloticus* Erichson, 1840**

**Material examined. Sajid Island**: Sajid, 16°45'42.6"N, 41°59'56.0"E, 6 m, 5.iii.2017, 2 (LT); 10.vi.2017, 3 (LT); 10.xi.2017, 9 (LT).

**Literature records.** Riyadh (Abdel-Dayem et al. 2017).

**General distribution.** AFR_ORR_SAR_SJP species, that is sporadically distributed in the Sub-Saharan Africa, North Africa east to Levant, Arabian Peninsula (KSA); from India to Japan.

**Remarks.** The species was collected by LT in sand dunes. Identification by A.A. Elgharbawy.

**137. *Bledius unicornis* (Germar, 1825)**

**Material examined. Sajid Island**: Sajid, 16°45'42.6"N, 41°59'56.0"E, 6 m, 10.vi.2017, 1 (LT).

**Literature records.** Eastern Province (Gusarov 1997).

**General distribution.** AFR_AUS_PAL_SAR species, widely distributed in North Africa and Europe; cited from Central Asia, Levant, Arabian Peninsula (KSA) and Australia.

**Remarks.***Bledius unicornis* was previously recorded from the Arabian Gulf coast (Gusarov 1997). Our unique specimen was collected by LT in a sand dune. Identification by A.A. Elgharbawy.

**138. *Cafius* sp.**

**Material examined. Sajid Island**: Sajid, 16°45'42.6"N, 41°59'56.0"E, 6 m, 13.x.2017, 10 (LT).

**Remarks.** The specimens were collected by LT in sand dunes.

**139. *Cafius* sp.**

**Material examined. Sajid Island**: Sajid, 16°45'42.6"N, 41°59'56.0"E, 6 m, 10.vi.2017, 73 (LT); 13.x.2017, 3 (LT); 10.xi.2017, 2 (LT); 2.xii.2017, 1 (LT).

**Remarks.** This unidentified species was collected by LT in sand dunes.

**140. *Ctenisomorphus* sp.**

**Material examined. Sajid Island**: Sajid, 16°45'42.6"N, 41°59'56.0"E, 6 m, 10.xi.2017, 1 (LT).

**Remarks.** The single listed specimen was collected by LT in a sand dune.

**141. *Enoptostomus* sp.**

**Material examined. Sajid Island**: Sajid, 16°45'42.6"N, 41°59'56.0"E, 6 m, 13.x.2017, 1 (LT).

**Remarks.** The unique species was collected by LT in a sand dune.

**142. *Philonthus* sp.**

**Material examined. Sajid Island**: Sajid, 16°45'42.6"N, 41°59'56.0"E, 6 m, 10.vi.2017, 2 (LT).

**Remarks.** This unidentified species was collected by LT in sand dunes.

**143. *Philonthus* sp.**

**Material examined. Sajid Island**: Sajid, 16°45'42.6"N, 41°59'56.0"E, 6 m, 10.vi.2017, 1 (LT).

**Remarks.** The single specimen, separable from the previous species, was collected by LT in a sand dune.

#### Superfamily Tenebrionoidea

**Family Anthicidae**

**144. *Anthelephila ionica* (LaFerté-Sénectère, 1849)**

**Material examined. Sajid Island**: Bridge, 16°51'27.4"N, 41°55'49.0"E, 2 m, 28.i.2017, 1 (SW). Sajid, 16°45'42.6"N, 41°59'56.0"E, 6 m, 28.i.2017, 3 (SW).

**Literature records.** This species was recorded as *Formicomus ionicus* Laferté, 1847 from Jizan (Uhmann 1998).

**General distribution.** PAL_SAR species, distributed in southern Europe; through Sahara from Libya east to Egypt and Levant, Arabia Peninsula (KSA, Yemen).

**Remarks.** The specimens of this species were collected by SW on *Dic. foveolatum* and *Ind. oblongifolia* in sand dunes. Identification by A. El-Torkey.

**145. *Anthelephila pilitarsis* Kejval, 2012**

**Material examined. Sajid Island**: Bridge, 16°51'27.4"N, 41°55'49.0"E, 2 m, 27.i.2017, 1 (SW); 18.ii.2017, 1 (SW). Sajid, 16°45'42.6"N, 41°59'56.0"E, 6 m, 25.i.2017, 5 (LT); 27.i.2017, 3 (SW); 18.ii.2017, 2 (LT); 5.iii.2017, 1 (LT); 2.iv.2017, 2 (LT); 13.x.2017, 5 (LT); 10.xi.2017, 20 (LT); 2.xii.2017, 25 (LT).

**General distribution.** Perhaps AFR species that was recently described from Socotra Island (Yemen). New country record for KSA.

**Remarks.** The species was frequently found in sand dunes and collected LT and by SW on *Dic. foveolatum* and *Ind. oblongifolia*. Identification by A. El-Torkey.

**146. *Anthicus crinitus* LaFerté-Sénectère, 1848**

**Material examined. Sajid Island**: Sajid, 16°45'42.6"N, 41°59'56.0"E, 6 m, 25.i.2017, 1 (LT); 2.xii.2017, 1 (LT).

**Literature records.** Asir, Baha, Makkah, Qaseem, and Riyadh (Abdel-Dayem et al. 2017).

**General distribution.**COS species.

**Remarks.** The above-listed specimens were collected by LT in sand dunes. Identification by A. El-Torkey.

**147. *Aulacoderus sefrensis* (Pic, 1894)**

**Material examined. Sajid Island**: Khotp, 16°52'47.6"N, 41°54'39.7"E, 11 m, 25.i.2017, 2 (SW). Sajid, 16°45'42.6"N, 41°59'56.0"E, 6 m, 25.i.2017, 1 (SW).

**General distribution.**SAR species, widely distributed from Morocco and Mauritania east to Egypt and Sinai. New country record for KSA.

**Remarks.** The species was collected by SW on *Ind. oblongifolia* and *Sen. alexandrina* in limestone areas and sand dunes, respectively. Identification by A. El-Torkey.

**148. *Cyclodinus debilis* (LaFerté-Sénectère, 1849)**

**Material examined. Sajid Island**: Sajid, 16°45'42.6"N, 41°59'56.0"E, 6 m, 10.xi.2017, 1 (LT).

**Literature records.** Eastern Province, Riyadh, and Tabouk (Uhmann 1998).

**General distribution.** ORR_PAL_SAR species, widely distributed throughout the Sahara Desert and North Africa east to Levant, Arabian Peninsula (KSA, Kuwait, United Arab Emirates, Yemen), and Iran to India; and northwards from southern Europe to central Asia.

**Remarks.** The unique listed specimen was collected by LT in a sand dune. Identification by A. El-Torkey.

**149. *Stricticollis modestus* (LaFerté-Sénectère, 1849)**

**Material examined. Sajid Island**: Khotp, 16°52'27.9"N, 41°54'43.9"E, 9 m, 28.i.2017, 1 (SW).

**Literature records.** Baha and Makkah (Uhmann 1998).

**General distribution.**SAR species, distributed from Morocco through North Africa, east to Levant and Arabian Peninsula (KSA, Oman, United Arab Emirates, Yemen), and extending northwards to Turkey and Kazakhstan.

**Remarks.** The single listed specimen was collected by SW on *Ind. oblongifolia* in a limestone area. Identification by A. El-Torkey.

#### Family Meloidae

**150. *Hycleus arabicus* (Pallas, 1781)**

**Material examined. Sajid Island**: Khotp, 16°52'47.6"N, 41°54'39.7"E, 11 m, 25.i.2017, 1 (SW).

**Literature records.** Jizan (Schneider 1991).

**General distribution.** END_AR species, cited from KSA and Yemen.

**Remarks.** This single listed specimen was collected by SW on *Ind. oblongifolia* in a limestone area. Identification by M. Bologna.

**151. *Lydomorphus* sp.**

**Material examined. Sajid Island**: Sajid, 16°45'42.6"N, 41°59'56.0"E, 6 m, 25.i.2017, 4 (SW).

**Remarks.** This unidentified species was collected in sand dunes by SW on *Sen. alexandrina*.

#### Family Mycetophagidae

**152. *Typhea stercorea* (Linnaeus, 1758)**

**Material examined. Sajid Island**: Sajid, 16°45'42.6"N, 41°59'56.0"E, 6 m, 10.vi.2017, 6 (LT); 13.x.2017, 5 (LT); 2.xii.2017, 1 (LT).

**Literature records.** Baha and Riyadh (Abdel-Dayem et al. 2017).

**General distribution.**COS species.

**Remarks.** The specimens of this species were collected by LT in sand dunes. Identification by H.H. Fadl.

#### Family Oedemeridae

**153. *Alloxantha* sp.**

**Material examined. Sajid Island**: Sajid, 16°45'42.6"N, 41°59'56.0"E, 6 m, 25.i.2017, 2 (SW); 5.iii.2017, 1 (LT).

**Remarks.** This species was collected by SW on *Sen. alexandrina* and by LT in sand dunes.

**154. *Probosca fuscipennis* (Blair, 1923)**

**Material examined. Sajid Island**: Sajid, 16°45'42.6"N, 41°59'56.0"E, 6 m, 25.i.2017, 1 (LT); 25.i.2017, 2 (SW); 5.iii.2017, 2 (LT); 3.xii.2017, 5 (LT).

**Literature records.** This taxon was reported under the genus *Ananconia* Seidlitz, 1899 from Eastern Province (Švihla 1980, 1984) and then transferred to the genus *Probosca* W. L. E. Schmidt, 1846 (Švihla 1990).

**General distribution.**SAR species, distributed in the Arabian Peninsula (KSA, Kuwait, United Arab Emirates), Iraq and Iran.

**Remarks.** The listed specimens were collected by SW on *Sen. alexandrina* and by LT in sand dunes. Identification by H.H. Fadl.

#### Family Scraptiidae

**155. *Anaspis* sp.**

**Material examined. Sajid Island**: Sajid, 16°45'42.6"N, 41°59'56.0"E, 6 m, 25.i.2017, 1 (LT); 25.i.2017, 2 (SW); 10.iv.2017, 3 (LT); 28.vii.2017, 1 (LT); 13.x.2017, 4 (LT); 10.xi.2017, 4 (LT); 2.xii.2017, 1 (LT).

**Remarks.** This species was frequently found in sand dunes and collected by SW on *Sen. alexandrina* and by LT.

**156. *Anaspis* sp.**

**Material examined. Sajid Island**: Khotp, 16°52'47.6"N, 41°54'39.7"E, 11 m, 25.i.2017, 3 (SW).

**Remarks.** This unidentified species was collected by SW on *Ind. oblongifolia* in limestone area.

#### Family Tenebrionidae

**157. *Adesmia interrupta* (Klug, 1830)**

**Material examined. Farasan Island**: Al-Huseis, 16°45'20.8"N, 42°03'59.6"E, 7 m, 25.i.2017, 6 (PT); 17.ii.2017, 5 (PT); 5.iii.2017, 33 (PT); 10.iv.2017, 51 (PT); 23.v.2017, 15 (PT); 10.vi.2017, 9 (PT); 28.vii.2017, 4 (PT); 6.ix.2017, 1 (PT); 13.x.2017, 2 (PT); 10.xi.2017, 0 (PT). Al-Kosar, 16°40'14.8"N, 42°08'54.9"E, 7 m, 25.i.2017, 4 (PT); 17.ii.2017, 6 (PT); 5.iii.2017, 28 (PT); 10.iv.2017, 51 (PT); 23.v.2017, 20 (PT); 10.vi.2017, 13 (PT); 28.vii.2017, 9 (PT); 11.viii.2017, 2 (PT); 13.x.2017, 3 (PT); 10.xi.2017, 1 (PT). Farasan Reserve, 16°42'26.3"N, 42°03'59.4"E, 5 m, 25.i.2017, 5 (PT); 17.ii.2017, 8 (PT); 5.iii.2017, 36 (PT); 10.iv.2017, 116 (PT); 23.v.2017, 120 (PT); 10.vi.2017, 40 (PT); 28.vii.2017, 4 (PT); 11.viii.2017, 2 (PT); 6.ix.2017, 9 (PT); 13.x.2017, 3 (PT); 10.xi.2017, 4 (PT). Farasan Reserve, 16°42'26.5"N, 42°04'19.2"E, 7 m, 25.i.2017, 6 (PT); 17.ii.2017, 6 (PT); 5.iii.2017, 135 (PT); 10.iv.2017, 98 (PT); 23.v.2017, 108 (PT); 10.vi.2017, 77 (PT); 28.vii.2017, 6 (PT); 6.ix.2017, 8 (PT); 13.x.2017, 2 (PT); 10.xi.2017, 5 (PT). **Sajid Island**: Bridge, 16°51'27.4"N, 41°55'49.0"E, 2 m, 25.i.2017, 5 (PT); 18.ii.2017, 11 (PT); 5.iii.2017, 50 (PT); 10.iv.2017, 60 (PT); 23.v.2017, 35 (PT); 10.vi.2017, 20 (PT); 28.vii.2017, 36 (PT); 12.viii.2017, 30 (PT); 6.ix.2017, 29 (PT); 13.x.2017, 7 (PT); 10.xi.2017, 5 (PT); 3.xii.2017, 3 (PT). Khotp, 16°52'27.9"N, 41°54'43.9"E, 9 m, 25.i.2017, 6 (PT); 18.ii.2017, 10 (PT); 5.iii.2017, 21 (PT); 10.iv.2017, 17 (PT); 23.v.2017, 12 (PT); 10.vi.2017, 8 (PT); 28.vii.2017, 6 (PT); 6.ix.2017, 7 (PT); 13.x.2017, 5 (PT); 10.xi.2017, 1 (PT); 3.xii.2017, 2 (PT). Khotp, 16°52'47.6"N, 41°54'39.7"E, 11 m, 25.i.2017, 5 (PT); 18.ii.2017, 4 (PT); 5.iii.2017, 63 (PT); 10.iv.2017, 27 (PT); 23.v.2017, 14 (PT); 10.vi.2017, 9 (PT); 28.vii.2017, 13 (PT); 6.ix.2017, 8 (PT); 13.x.2017, 3 (PT); 10.xi.2017, 2 (PT); 3.xii.2017, 1 (PT). Sajid, 16°45'42.6"N, 41°59'56.0"E, 6 m, 25.i.2017, 8 (PT); 18.ii.2017, 6 (PT); 5.iii.2017, 50 (PT); 10.iv.2017, 40 (PT); 23.v.2017, 35 (PT); 10.vi.2017, 12 (PT); 28.vii.2017, 50 (PT); 6.ix.2017, 3 (PT); 13.x.2017, 2 (PT); 10.xi.2017, 2 (PT); 3.xii.2017, 3 (PT).

**Literature records.** Makkah (Kaszab 1981).

**General distribution.** END_AR species, a southwestern Arabian Peninsula endemic (KSA, Yemen).

**Remarks.** This was the most common species of the family that occurred in all habitats sampled and throughout the year. Identification by M.S. Abdel-Dayem.

**158. *Alphitobius diaperinus* (Panzer, 1797)**

**Material examined. Sajid Island**: Sajid, 16°45'42.6"N, 41°59'56.0"E, 6 m, 28.iv.2017, 1 (LT); 2.xii.2017, 4 (LT).

**Literature records.** Jizan, Makkah, Najran, Qaseem, and Riyadh (Kaszab 1979, 1982).

**General distribution.**COS species.

**Remarks.** This species was collected by LT in sand dunes. Identification by A. El-Torkey and W. Schawaller.

**159. *Alphitobius laevigatus* (Fabricius, 1781)**

**Material examined. Sajid Island**: Sajid, 16°45'42.6"N, 41°59'56.0"E, 6 m, 25.i.2017, 1 (LT).

**Literature records.** Eastern Province, Madinah, Makkah, and Riyadh (Kaszab 1979, 1982).

**General distribution.**COS species.

**Remarks.** The unique listed specimen was collected by LT in a sand dune. Identification by A. El-Torkey and W. Schawaller.

**160. *Cheirodes brevicollis* (Wollaston, 1864)**

**Material examined. Sajid Island**: Sajid, 16°45'42.6"N, 41°59'56.0"E, 6 m, 5.iii.2017, 1 (LT); 10.vi.2017, 1 (LT); 13.x.2017, 3 (LT); 10.xi.2017, 2 (LT).

**Literature records.** Asir, Baha, Eastern Province, Hail, Jizan, Makkah, and Riyadh (Kaszab 1979, 1982; El-Hawagry et al. 2013).

**General distribution.** AFR_ORR_PAL_SAR species, distributed through the entire Sahara Desert, from Morocco to Pakistan, north to southern Europe and Central Asia; cited from East Africa and China.

**Remarks.** The species was collected by LT in sand dunes. Identification by A. El-Torkey and W. Schawaller.

**161. *Cheirodes sardous* Gené, 1839**

**Material examined. Sajid Island**: Sajid, 16°45'42.6"N, 41°59'56.0"E, 6 m, 10.vi.2017, 2 (LT); 2.xii.2017, 1 (LT).

**Literature records.** Asir, Eastern Province, Hail, Jizan, Madinah, Makkah, Najran, Riyadh, and Tabouk (Kaszab 1979, 1982).

**General distribution.** AUS_PAL_SAR species, distributed in North Africa, east to Levant, Arabian Peninsula (KSA, United Arab Emirates) and Iran, north to southern and western Europe, and cited from Australia.

**Remarks.** This species was collected by LT in sand dunes. Identification by A. El-Torkey and W. Schawaller.

**162. *Clitobius cribricollis* (Allard, 1882)**

**Material examined. Farasan Island**: Al-Huseis, 16°45'20.8"N, 42°03'59.6"E, 7 m, 10.vi.2017, 1 (PT); 13.x.2017, 1 (PT). Al-Kosar, 16°40'14.8"N, 42°08'54.9"E, 7 m, 5.iii.2017, 1 (PT). Farasan Reserve, 16°42'26.5"N, 42°04'19.2"E, 7 m, 12.viii.2017, 2 (PT). **Sajid Island**: Bridge, 16°51'27.4"N, 41°55'49.0"E, 2 m, 10.vi.2017, 3 (PT); 3.xii.2017, 2 (PT). Khotp, 16°52'27.9"N, 41°54'43.9"E, 9 m, 23.v.2017, 1 (PT). Sajid, 16°45'42.6"N, 41°59'56.0"E, 6 m, 17.ii.2017, 5 (PT); 10.iv.2017, 4 (LT); 10.iv.2017, 2 (PT); 10.vi.2017, 1 (LT); 6.ix.2017, 1 (LT).

**General distribution.**AFR species, known from East Africa (Ethiopia) and Arabian Peninsula (Yemen). New country record for KSA.

**Remarks.** The collected specimens were found in coastal sandy areas, limestone areas, salt marshes, and sand dunes. Identification by W. Schawaller.

**163. *Diphyrrhynchus aenescens* (Fairmaire, 1892)**

**Material examined. Sajid Island**: Sajid, 16°45'42.6"N, 41°59'56.0"E, 6 m, 17.ii.2017, 2 (LT); 5.iii.2017, 1 (LT); 28.iv.2017, 3 (LT); 10.vi.2017, 2 (LT); 10.xi.2017, 40 (LT); 2.xii.2017, 2 (LT).

**Literature records.** Jizan and Makkah (Kaszab 1982; Schawaller 1991).

**General distribution.**AFR species, distributed in Egypt (Sinai), Arabian Peninsula (KSA, Yemen), Madagascar.

**Remarks.** The above-listed specimens were frequently collected in sand dunes by LT. Identification by W. Schawaller.

**164. *Gonocephalum patruele* (Erichson, 1843)**

**Material examined. Farasan Island**: Al-Huseis, 16°45'20.8"N, 42°03'59.6"E, 7 m, 17.ii.2017, 1 (PT); 23.v.2017, 2 (PT). Farasan Reserve, 16°42'26.3"N, 42°03'59.4"E, 5 m, 17.ii.2017, 2 (PT); 12.viii.2017, 1 (PT). Farasan Reserve, 16°42'26.5"N, 42°04'19.2"E, 7 m, 17.ii.2017, 1 (PT); 5.iii.2017, 9 (PT); 13.x.2017, 1 (PT). **Sajid Island**: Bridge, 16°51'27.4"N, 41°55'49.0"E, 2 m, 25.i.2017, 1 (PT); 18.ii.2017, 2 (PT). Khotp, 16°52'27.9"N, 41°54'43.9"E, 9 m, 25.i.2017, 4 (PT); 18.ii.2017, 5 (PT); 5.iii.2017, 8 (PT); 5.iii.2017, 5 (PT); 23.v.2017, 5 (PT); 10.vi.2017, 1 (PT); 12.viii.2017, 2 (PT); 13.x.2017, 13 (PT); 10.xi.2017, 2 (PT); 3.xii.2017, 3 (PT). Khotp, 16°52'47.6"N, 41°54'39.7"E, 11 m, 25.i.2017, 2 (PT); 18.ii.2017, 3 (PT); 23.v.2017, 4 (PT); 13.x.2017, 3 (PT); 3.xii.2017, 2 (PT). Sajid, 16°45'42.6"N, 41°59'56.0"E, 6 m, 25.i.2017, 5 (PT); 18.ii.2017, 4 (PT); 5.iii.2017, 1 (PT); 10.iv.2017, 62 (PT); 12.viii.2017, 8 (PT); 6.ix.2017, 5 (PT); 13.x.2017, 5 (LT); 13.x.2017, 9 (PT); 10.xi.2017, 5 (LT); 10.xi.2017, 21 (PT); 3.xii.2017, 4 (PT).

**Literature records.** Hail, Jouf, Qaseem, and Riyadh (Kaszab 1979, 1982).

**General distribution.** AFR_SAR species, distributed in Sub-Saharan Africa, north to North Africa, from Canary Islands and Morocco eastward to Levant, Arabian Peninsula (KSA, Oman, United Arab Emirates, Yemen), Iran and Afghanistan.

**Remarks.** This species was found in coastal sandy areas, limestone areas, salt marshes and sand dunes. Identification by A. El-Torkey and W. Schawaller.

**165. *Gonocephalum prolixum* (Erichson, 1843)**

**Material examined. Sajid Island**: Sajid, 16°45'42.6"N, 41°59'56.0"E, 6 m, 6.ix.2017, 1 (LT); 2.xii.2017, 1 (LT).

**General distribution.**AFR species, widely distributed in Saharan and Sub-Saharan Africa, reaches its northern limits at Cyprus, Italy, Rhodes, and Spain, and extended eastwardly to Levant, Arabian Peninsula (KSA, United Arab Emirates), and Iran. Identification by A. El-Torkey and W. Schawaller.

**Literature records.** Asir, Madinah, Makkah, and Najran (Kaszab 1979, 1982).

**Remarks.** This species was collected by LT in sand dunes.

**166. *Gonocephalum rusticum* (Olivier, 1811)**

**Material examined. Sajid Island**: Sajid, 16°45'42.6"N, 41°59'56.0"E, 6 m, 10.vi.2017, 1 (LT); 3.xii.2017, 7 (LT).

**Literature records.** Eastern Province, Makkah, Qaseem, and Riyadh (Kaszab 1979, 1982).

**General distribution.** PAL_SAR species, distributed through Sahara Desert, from Morocco east to Levant, Arabian Peninsula (KSA, Oman), Iran and Afghanistan; north to southern Europe and Central Asia. There is a record from China.

**Remarks.** The above-listed specimens were collected by LT in sand dunes. Identification by A. El-Torkey and W. Schawaller.

**167. *Hymenalia denticulata* (Muche, 1982)**

**Material examined. Sajid Island**: Sajid, 16°45'42.6"N, 41°59'56.0"E, 6 m, 25.i.2017, 2 (LT); 26.i.2017, 1 (SW); 10.iv.2017, 2 (LT); 3.xii.2017, 2 (LT).

**Literature records.** Baha, Makkah, and Riyadh (Muche 1982).

**General distribution.** END_AR species, distributed only in the Arabian Peninsula (KSA, Oman, United Arab Emirates).

**Remarks.** The above specimens were collected by SW on by *Sen. alexandrina* and by LT in sand dunes. Identification by W. Schawaller.

**168. *Imatismus villosus* (Haag-Rutenberg, 1870)**

**Material examined. Farasan Island**: Al-Huseis, 16°45'20.8"N, 42°03'59.6"E, 7 m, 5.iii.2017, 2 (SW); 6.ix.2017, 1 (SW); 3.xii.2017, 1 (SW). Farasan Reserve, 16°42'26.3"N, 42°03'59.4"E, 5 m, 10.iv.2017, 1 (SW). Farasan Reserve, 16°42'26.5"N, 42°04'19.2"E, 7 m, 5.iii.2017, 2 (SW); 6.ix.2017, 1 (SW). **Sajid Island**: Bridge, 16°51'27.4"N, 41°55'49.0"E, 2 m, 12.ii.2017, 1 (SW); 12.ii.2017, 1 (SW); 5.iii.2017, 2 (PT); 10.iv.2017, 1 (SW); 5.v.2017, 1 (SW). Khotp, 16°52'27.9"N, 41°54'43.9"E, 9 m, 10.iv.2017, 1 (SW). Khotp, 16°52'47.6"N, 41°54'39.7"E, 11 m, 12.ii.2017, 1 (SW); 5.iii.2017, 1 (SW). Sajid, 16°45'42.6"N, 41°59'56.0"E, 6 m, 12.ii.2017, 1 (SW); 5.iii.2017, 2 (SW); 10.iv.2017, 16 (SW); 28.vii.2017, 1 (LT); 12.viii.2017, 2 (SW); 13.x.2017, 3 (SW); 5.xi.2017, 2 (SW).

**Literature records.** Asir, Baha, and Makkah (Kaszab 1979, 1982).

**General distribution.** AFR_ORR_SAR species, distributed sporadically in Sub-Saharan Africa and throughout most of the Sahara Desert, from Morocco east to Levant, Arabian Peninsula (KSA, Oman, United Arab Emirates, Yemen), Iran Pakistan, India; reaches its north limits at southern Europe (Italy, Greece, Rhodes).

**Remarks.** The listed specimens were collected by SW on *Ble. edulis* and *Tet. alba* in salt marshes, *Dic. foveolatum* and *Ind. oblongifolia* in sand dunes, *Lim. axillare* in coastal sandy areas, and *Aer. javanica*, *Ind. spinosa*, and *Sen. alexandrina* in limestone areas. Also, specimens were collected by PT under the canopy of *Abu. pannosum* and by LT in sand dunes. Identification by W. Schawaller.

**169. *Mycetocharina rufofusca* Muche, 1982**

**Material examined. Sajid Island**: Sajid, 16°45'42.6"N, 41°59'56.0"E, 6 m, 25.i.2017, 1 (LT); 10.iv.2017, 2 (LT); 3.xii.2017, 2 (LT).

**Literature records.** Asir, Baha, Makkah, and Riyadh (Muche 1982).

**General distribution.** END_SA species, known only from KSA.

**Remarks.** The species was collected by LT in sand dunes. Identification by A.A. Elgharbawy and W. Schawaller.

**170. *Opatroides punctulatus* Brullé, 1832**

**Material examined. Farasan Island**: Farasan Reserve, 16°42'26.3"N, 42°03'59.4"E, 5 m, 25.i.2017, 1 (PT). **Sajid Island**: Sajid, 16°45'42.6"N, 41°59'56.0"E, 6 m, 13.x.2017, 1 (SW).

**Literature records.** Eastern Province, Jouf, Madinah, Makkah, Qaseem, and Riyadh (Kaszab 1979, 1982).

**General distribution.** AFR_ORR_PAL_SAR, distributed in the Sahara Desert, from Morocco eastwardly to Arabian Peninsula (KSA, Qatar, Yemen), Iran Pakistan and reaches the Indian Subcontinent; north to southern Europe and Central Asia; south to East Africa.

**Remarks.** The above-listed specimens were collected by SW on *Ble. edulis* and *Sen. alexandrina* in salt marshes and sand dunes, respectively. Identification by A. El-Torkey and W. Schawaller.

**171. *Oxycara dhofarensis* Lillig, 2009**

**Material examined. Farasan Island**: Al-Huseis, 16°45'20.8"N, 42°03'59.6"E, 7 m, 17.ii.2017, 3 (PT); 5.iii.2017, 20 (PT); 10.iv.2017, 6 (PT); 23.v.2017, 15 (PT); 10.vi.2017, 1 (PT); 28.vii.2017, 2 (PT); 11.viii.2017, 2 (PT); 5.ix.2017, 1 (PT); 13.x.2017, 3 (PT); 3.xii.2017, 4 (PT). Al-Kosar, 16°40'14.8"N, 42°08'54.9"E, 7 m, 25.i.2017, 1 (PT); 17.ii.2017, 2 (PT); 5.iii.2017, 14 (PT); 5.iii.2017, 6 (PT); 10.iv.2017, 5 (PT); 23.v.2017, 35 (PT); 10.vi.2017, 9 (PT); 11.viii.2017, 8 (PT); 5.ix.2017, 4 (PT). Farasan Reserve, 16°42'26.3"N, 42°03'59.4"E, 5 m, 17.ii.2017, 4 (PT); 10.iv.2017, 29 (PT); 23.v.2017, 12 (PT); 10.vi.2017, 25 (PT); 28.vii.2017, 6 (PT); 11.viii.2017, 1 (PT); 5.ix.2017, 1 (PT); 13.x.2017, 2 (PT). Farasan Reserve, 16°42'26.5"N, 42°04'19.2"E, 7 m, 25.i.2017, 1 (PT); 17.ii.2017, 3 (PT); 5.iii.2017, 30 (PT); 10.iv.2017, 86 (PT); 23.v.2017, 35 (PT); 10.vi.2017, 1 (PT); 28.vii.2017, 12 (PT); 11.viii.2017, 1 (PT); 5.ix.2017, 1 (PT). **Sajid Island**: Bridge, 16°51'27.4"N, 41°55'49.0"E, 2 m, 25.i.2017, 2 (PT); 18.ii.2017, 6 (PT); 3.iii.2017, 9 (LT); 5.iii.2017, 10 (PT); 10.iv.2017, 19 (PT); 23.v.2017, 8 (PT); 10.vi.2017, 5 (PT); 12.viii.2017, 25 (PT); 6.ix.2017, 14 (PT); 13.x.2017, 38 (PT); 3.xii.2017, 1 (PT). Khotp, 16°52'27.9"N, 41°54'43.9"E, 9 m, 25.i.2017, 4 (PT); 18.ii.2017, 4 (PT); 10.iv.2017, 5 (PT); 23.v.2017, 4 (PT); 10.vi.2017, 2 (PT); 12.viii.2017, 3 (PT); 13.x.2017, 3 (PT); 3.xii.2017, 2 (PT). Khotp, 16°52'47.6"N, 41°54'39.7"E, 11 m, 25.i.2017, 3 (PT); 18.ii.2017, 7 (PT); 10.iv.2017, 12 (PT); 23.v.2017, 12 (PT); 10.vi.2017, 6 (PT); 11.viii.2017, 3 (PT); 10.xi.2017, 3 (PT); 3.xii.2017, 2 (PT). Sajid, 16°45'42.6"N, 41°59'56.0"E, 6 m, 25.i.2017, 2 (PT); 18.ii.2017, 3 (PT); 5.iii.2017, 2 (PT); 10.iv.2017, 18 (PT); 10.vi.2017, 7 (PT); 12.viii.2017, 5 (PT); 5.ix.2017, 1 (LT); 6.ix.2017, 3 (PT); 10.xi.2017, 20 (PT); 3.xii.2017, 2 (PT).

**General distribution.** END_AR species, recently described from Oman. New country record for KSA.

**Remarks.** The specimens of this species were collected from different habitats throughout the year. Identification by M.S. Abdel-Dayem.

**172. *Palorus subdepressus* (Wollaston, 1864)**

**Material examined. Sajid Island**: Sajid, 16°45'42.6"N, 41°59'56.0"E, 6 m, 25.i.2017, 2 (SW); 10.vi.2017, 34 (LT); 13.x.2017, 4 (LT).

**Literature records.** Eastern Province, Makkah, Qaseem, and Riyadh (Kaszab 1979, 1982).

**General distribution.**COS species.

**Remarks.** This species was collected by SW on *Sen. alexandrina* and by LT in sand dunes. Identification by W. Schawaller.

**173. *Phaleria prolixa* Fairmaire, 1869**

**Material examined. Sajid Island**: Sajid, 16°45'42.6"N, 41°59'56.0"E, 6 m, 2.xii.2017, 1 (LT).

**Literature records.** Eastern Province and Najran (Kaszab 1982).

**General distribution.** AFR_SAR species, distributed in East Africa, Egypt, the entire Arabian Peninsula, Iran.

**Remarks.** The unique listed above specimen was collected by LT in a sand dune. Identification by W. Schawaller.

**174. *Phtora subclavata* (Wollaston, 1861)**

**Material examined. Sajid Island**: Khotp, 16°52'27.9"N, 41°54'43.9"E, 9 m, 10.xi.2017, 1 (PT). Sajid, 16°45'42.6"N, 41°59'56.0"E, 6 m, 25.i.2017, 1 (LT); 5.iii.2017, 6 (LT); 28.iv.2017, 4 (LT); 23.v.2017, 2 (LT); 13.x.2017, 2 (LT); 10.xi.2017, 10 (LT); 2.xii.2017, 1 (LT).

**Literature records.** Eastern Province and Riyadh (Kaszab 1979).

**General distribution.**SAR species, distributed in Egypt (including Sinai), Israel, Arabian Peninsula (KSA).

**Remarks.** The above-listed specimens were collected by PT under the canopy of *Ind. oblongifolia* in limestone area and by LT in sand dunes. Identification by W. Schawaller.

**175. *Sclerum orientale* (Fabricius, 1775)**

**Material examined. Sajid Island**: Khotp, 16°52'27.9"N, 41°54'43.9"E, 9 m, 18.ii.2017, 1 (PT). Sajid, 16°45'42.6"N, 41°59'56.0"E, 6 m, 10.iv.2017, 4 (PT); 13.x.2017, 1 (PT); 10.xi.2017, 1 (PT); 2.xii.2017, 1 (SW).

**Literature records.** Riyadh (Kaszab 1982).

**General distribution.** AFR_SAR species, distributed in East Africa (Sudan), east to the Arabian Peninsula (KSA, Yemen), north to Egypt and Levant.

**Remarks.** The above-listed specimens were collected by PT under the canopies of *Ind. oblongifolia* in limestone areas and under *Sen. alexandrina* sand dunes. Identification by A. El-Torkey and W. Schawaller.

**176. Tenebrio cf. angustus Zoufal, 1892**

**Material examined. Sajid Island**: Bridge, 16°51'27.4"N, 41°55'49.0"E, 2 m, 10.iv.2017, 3 (PT); 6.ix.2017, 1 (PT). Khotp, 16°52'27.9"N, 41°54'43.9"E, 9 m, 18.ii.2017, 2 (PT); 5.iii.2017, 2 (PT); 10.iv.2017, 1 (PT); 23.v.2017, 3 (PT); 10.vi.2017, 1 (PT); 3.xii.2017, 1 (PT). Sajid, 16°45'42.6"N, 41°59'56.0"E, 6 m, 25.i.2017, 1 (LT); 18.ii.2017, 2 (LT); 5.iii.2017, 2 (LT); 5.iii.2017, 2 (PT); 10.vi.2017, 1 (LT); 12.viii.2017, 1 (LT); 5.ix.2017, 4 (LT); 13.x.2017, 36 (LT); 10.xi.2017, 4 (LT); 3.xii.2017, 2 (LT).

**General distribution.** PAL_SAR species, distributed in the Central Asia and South Caucasus, extends southwardly to the Sahara Desert (Afghanistan, Iran). New country record for KSA.

**Remarks.** This species was collected by PT under the canopies of *Ind. oblongifolia* in limestone areas and under *Aer. javanica*, *Dic. foveolatum*, *Ind. oblongifolia*, and *Sen. alexandrina* in sand dunes. Identification by M.S. Abdel-Dayem.

**177. *Trachyderma philistina* Reiche & Saulcy, 1857**

**Material examined. Farasan Island**: Al-Huseis, 16°45'20.8"N, 42°03'59.6"E, 7 m, 10.iv.2017, 3 (PT); 23.v.2017, 1 (PT); 28.vii.2017, 1 (PT); 13.x.2017, 1 (PT); 10.xi.2017, 1 (PT). Al-Kosar, 16°40'14.8"N, 42°08'54.9"E, 7 m, 10.iv.2017, 8 (PT); 23.v.2017, 3 (PT); 10.vi.2017, 1 (PT); 11.viii.2017, 1 (PT); 10.xi.2017, 1 (PT). Farasan Reserve, 16°42'26.3"N, 42°03'59.4"E, 5 m, 10.iv.2017, 102 (PT); 23.v.2017, 1 (PT); 5.ix.2017, 9 (PT); 13.x.2017, 1 (PT); 10.xi.2017, 3 (PT). Farasan Reserve, 16°42'26.5"N, 42°04'19.2"E, 7 m, 10.iv.2017, 33 (PT); 23.v.2017, 3 (PT); 5.ix.2017, 7 (PT); 13.x.2017, 2 (PT); 10.xi.2017, 2 (PT). **Sajid Island**: Bridge, 16°51'27.4"N, 41°55'49.0"E, 2 m, 23.v.2017, 3 (PT); 28.vii.2017, 1 (PT); 6.ix.2017, 2 (PT). Khotp, 16°52'27.9"N, 41°54'43.9"E, 9 m, 23.v.2017, 1 (PT); 28.vii.2017, 3 (PT); 6.ix.2017, 1 (PT). Khotp, 16°52'47.6"N, 41°54'39.7"E, 11 m, 12.viii.2017, 1 (PT). Sajid, 16°45'42.6"N, 41°59'56.0"E, 6 m, 10.iv.2017, 1 (PT); 28.vii.2017, 1 (PT); 12.viii.2017, 1 (PT); 6.ix.2017, 4 (PT).

**Literature records.** Baha, Eastern Province, Hail, Makkah, Northern Frontier, Qaseem, Riyadh, and Tabouk (Kaszab 1982).

**General distribution.**SAR species, distributed from Egypt to Levant, Arabian Peninsula (KSA, Bahrain, Oman, United Arab Emirates, Yemen), Iran; extends northwards to southern Europe (Greece, Rhodes); recorded from the north of Sudan.

**Remarks.** The above-listed specimens were collected by PT under the canopies of *Lim. axillare* and *Sal. persica* in coastal sandy areas, *Ind. oblongifolia* in limestone areas, *Ble. edulis* and *Sua. monoica* in salt marshes, and *Aer. javanica*, *Ind. oblongifolia* and *Sen. alexandrina* sand dunes. Identification by M.S. Abdel-Dayem.

**178. *Zophosis bicarinata quadricostata* Solier, 1834**

**Material examined. Farasan Island**: Al-Huseis, 16°45'20.8"N, 42°03'59.6"E, 7 m, 23.v.2017, 3 (PT). **Sajid Island**: Bridge, 16°51'27.4"N, 41°55'49.0"E, 2 m, 10.vi.2017, 2 (PT); 6.ix.2017, 2 (PT). Khotp, 16°52'27.9"N, 41°54'43.9"E, 9 m, 5.ix.2017, 2 (SW). Sajid, 16°45'42.6"N, 41°59'56.0"E, 6 m, 10.iv.2017, 2 (PT); 12.viii.2017, 3 (PT); 5.ix.2017, 4 (PT); 6.ix.2017, 2 (PT); 10.xi.2017, 3 (PT).

**Literature records.** Makkah (Kaszab 1981).

**General distribution.**SAR species, distributed in Egypt, Levant, Arabian Peninsula (KSA, Yemen), Sudan.

**Remarks.** This subspecies is recorded as *Zophosis quadricostata* Solier from the Red Sea coast (Kaszab 1981). The above-listed specimens were collected by PT under the canopies of *Com. gileadensis* in coastal sandy areas, *Ind. oblongifolia* in limestone areas, and on *Aer. javanica*, *Ind. oblongifolia*, and *Sen. alexandrina* in sand dunes. Identification by M.S. Abdel-Dayem.

#### Family Zopheridae

**179. *Monomma* sp.**

**Material examined. Sajid Island**: Khotp, 16°52'27.9"N, 41°54'43.9"E, 9 m, 13.x.2017, 2 (PT). Sajid, 16°45'42.6"N, 41°59'56.0"E, 6 m, 18.ii.2017, 1 (LT); 3.xii.2017, 3 (LT).

**Remarks.** The specimens of this species were collected by PT under the canopy of *Ind. oblongifolia* in limestone areas and by LT in sand dunes.

## Discussion

**Figure 4.**
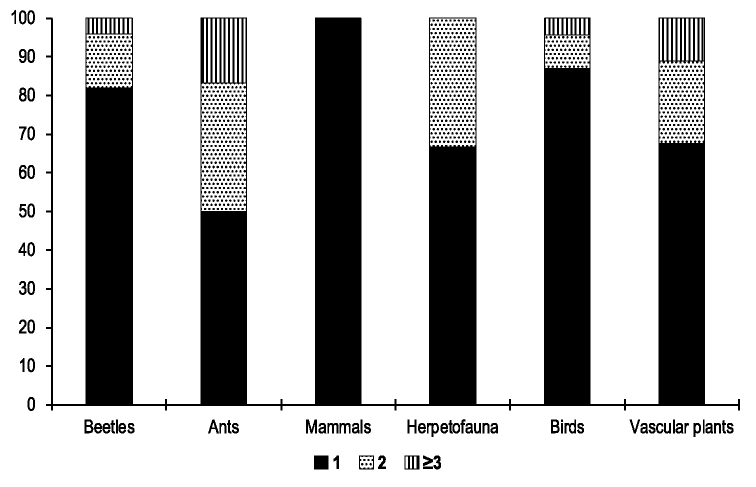
Proportion of genera represented in the Farasan Archipelago, Kingdom of Saudi Arabia by one (1), two (2), and three or more (> 3) species in Coleoptera (current data), Formicidae (based on Sharaf and Al-Zailaie 2006), mammals and herpetofauna (based on Masseti 2010, 2014), birds (based on Jennings 1988), and vascular plants (based on Alfarhan et al. 2002).

Our study is the first survey of the beetles of the Farasan Archipelago. We provide records for a total of 31 families, 145 genera, and 179 species of beetles. This survey resulted in new records for one genus, *Lasiocera*, and one species, *A. villiersanus* for the Arabian Peninsula, and 18 species for KSA. Five families collectively composed more than 50% of the total species richness, the Carabidae (31 spp., 17.3%), Tenebrionidae (22 spp., 12.3%), Chrysomelidae (17 spp., 9.5%), Scarabaeidae (13 spp., 7.3%), and Coccinellidae (12 spp., 6.7%). Most genera (82.1%) and 32.3% of the families were represented by a single species (Fig. 4). Other groups such as ants (Sharaf and Al-Zailaie 2006), mammals (Masseti 2010), herpetofauna (Masseti 2014), birds (Jennings 1988), and vascular plants (Alfarhan et al. 2002) demonstrate similar taxonomic diversity patterns. Sixteen beetle species (8.9%) were collected from all different habitats surveyed. Fifty-one percent of all species (92 spp.) occurred only in sand dune areas and 11.2% (20 spp.) only inhabited limestone areas, associated with sand areas. Only two species, *A. congregata* (Buprestidae) and *S. chalcites* (Histeridae), were collected from salt marshes. *Alphitobius diaperinus*, *A. laevigatus*, and *P. subdepressus* (Tenebrionidae) were the only recorded synanthropic species.

Farasan Al-Kabir Island had smaller number of species (34 spp.) than Sajid Island (176 spp.), despite its larger size. However, both islands shared 31 species. This may be due to the larger human population and higher level of human impacts on the environment of Farasan Island than of Sajed Island. Negative human influence on the biodiversity has been frequently documented (Mandura and Khafaji 1993; Van Damme and Banfield 2011; Bailey et al. 2007; Gillespie et al. 2008; Warren et al. 2015; McGill et al. 2015).

**Figure 5.**
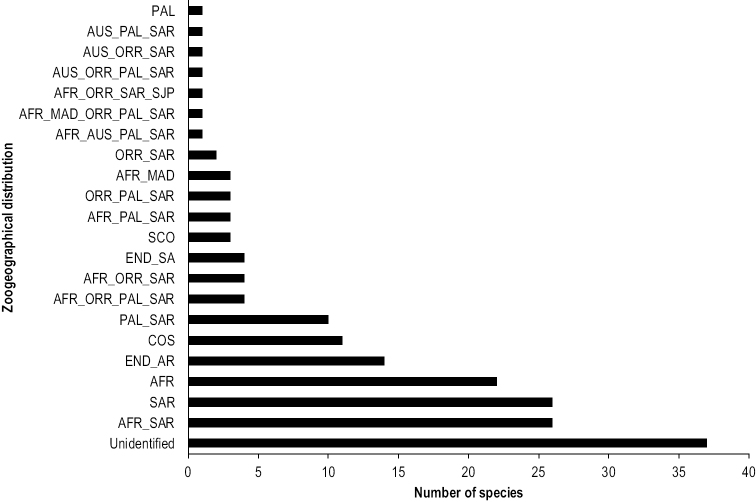
Zoogeographical composition of the beetle fauna of Farasan Archipelago, southern Red Sea, KSA, based on their general distribution. AFR, Afrotropical; AUS, Australian; COS, Cosmopolitan; MAD, Madagascan; ORR, Oriental; PAL, Palaearctic; SAR, Saharo-Arabian; SCO, Subcosmopolitan; SJP, Sino_Japanese.

The geographical location of Farasan Archipelago between the Arabian Peninsula, Africa, and southeast Asia undoubtedly affects the composition of its flora and fauna (El-Demerdash 1996; Tomas et al. 2010; Masseti 2014). Thus, it is not surprising that 41.3% of the species belong to Saharo-Arabian and Afrotropical faunal elements (Fig. 5). Oriental elements (2 spp., 1.1%) contributed a low number of species. The cosmopolitan and subcosmopolitan elements are represented by only 14 species (9.8%). Similar to the Red Sea archipelagos which show a scarcity of endemism in comparison to the Mediterranean Sea or the Indian Ocean (Miller and Nyberg 1991; Masseti 2010, 2014), the Farasan Archipelago apparently has no endemic beetle species. In contrast, the Archipelago harbors eighteen species (10.1%) endemic to the Arabian Peninsula (14 spp.) and KSA (4 spp.). This may be due to the fact that these territories were connected to the southwestern Arabian Peninsula at the end of the Pleistocene (Macfadyen 1930).

A direct comparison of the beetle fauna of KSA mainland with the beetles now known from Farasan Archipelago is problematic, since there are no available comprehensive lists of the beetles of KSA. Based on the published data on beetle distributions known from specific regions of KSA, more than half the Archipelago beetle species (64.8%, 116 spp.) also occur elsewhere on the KSA mainland. The Archipelago shares 91 species (50.8%) with southern and southwestern regions of KSA, 71 species (39.7%) with the central region, 32 species (17.9%) with eastern region, and 21 species (11.7%) with northern and northwestern regions of KSA. Similar relationships between the Archipelago and the KSA mainland have been reported for other faunas (Jennings 1988; Sharaf and Al-Zailaie 2006; Masseti 2010, 2014) and flora (El-Demerdash 1996, Tomas et al. 2010). The majority of species of beetles occurring on this Archipelago disperse from the mainland of Arabian shelf to the east and in the Eritrean Shelf to the west; this supports the concept that islands that are near the mainland will potentially receive propagules from more species than will distant islands (Rosenzweig 1995; Lövei et al. 2006; Warren et al. 2015).

Faunistic data are scarce for the beetles from the Red Sea islands and this makes the comparisons with these islands difficult. Although the Socotra Islands are more distant (1,357 km) in the Indian Ocean, and much larger (3,800 km^2^), they are the only nearest islands for comparison in which significant progress has been made in entomological studies over the last thirty years (Bezděk and Hájek 2017). The fauna of Socotra comprises 645 beetle species (Hájek and Bezděk 2019), ca. 3.5 times more species than recorded from Farasan Archipelago. The families of beetles recorded by us from Farasan Archipelago are also known from the Socotra Archipelago except for one family, the Glaresidae (Table 1). Seventy of the 145 genera (48.3%) on Farasan Archipelago are shared with the 343 genera on the Socotra Archipelago whereas only 28 of Farasan Archipelago’s 179 species (15.6%) are shared with the 645 species currently known from the Socotran fauna, half (50%, 14 spp.) of which are Saharo-Arabian and Afrotropical and 28.8% (8 spp.) are cosmopolitan and subcosmopolitan species. The long-term isolation (16–30 million years ago) and more detached location (approximately 240 km east of Africa and 380 km from Arabian Peninsula) of the Socotra islands have no doubt allowed the development of a unique fauna having a high proportion (47%, 305 spp.) of endemic taxa (Hájek and Bezděk 2019). In comparison, no endemic beetle species was recognized in the Farasan Islands, which have been detached from Arabian Peninsula for only ca. five million years (Bailey et al. 2015) and then only by 40 kilometers.

**Table 1.** Comparison of beetle families, genera and species shared between Farasan and Socotra archipelagos.

Family	Number of species (genera)		Number of Shared species (genera)	% Shared species (genera)
	Farasan	Socotra		
Aderidae	–	2	–	–
Anthicidae	6 (5)	8 (5)	2 (2)	1.1% (1.4%)
Anthribidae	–	2 (?)	–	–
Attelabidae	–	1 (1)	–	–
Belidae	–	1 (1)	–	–
Bostrichidae	4 (4)	9 (8)	–	(0.7%)
Bothrideridae	–	1 (?)	–	–
Brentidae	2 (2)	5 (4)	–	–
Buprestidae	4 (4)	10 (5)	–	(0.7%)
Cantharidae	–	2 (2)	–	–
Carabidae	31 (25)	59 (37)	6 (16)	3.4% (11%)
Cerambycidae	2 (2)	17 (14)	1 (2)	0.6% (1.4%)
Chrysomelidae	17 (15)	56 (31)	4 (8)	2.2% (5.5%)
Ciidae	–	2 (?)	–	–
Cleridae	4 (4)	5 (3)	1 (1)	0.6% (0.7%)
Coccinellidae	12 (8)	14 (10)	2 (5)	1.1% (3.4%)
Corylophidae	–	1 (?)	–	–
Curculionidae	9 (8)	76 (33)	–	(0.7%)
Cybocephalidae	–	2 (?)	–	
Dermestidae	6 (3)	20 (6)	1 (3)	0.6% (2.1%)
Dytiscidae	4 (2)	14 (10)	3 (2)	1.7% (1.4%)
Elateridae	9 (9)	26 (12)	(4)	% (2.8%)
Erotylidae	–	1 (?)	–	–
Georissidae	–	3 (1)	–	–
Geotrupidae	–	2 (1)	–	–
Glaresidae	1 (1)	–	–	–
Gyrinidae	–	3 (2)	–	–
Heteroceridae	–	2 (2)	–	–
Histeridae	3 (1)	21 (12)	1 (1)	(0.7%)
Hybosoridae	1 (1)	1 (1)	1 (1)	(0.7%)
Hydraenidae	–	2 (2)	–	–
Hydrophilidae	2 (1)	13 (10)	1 (1)	(0.7%)
Laemophloeidae	1 (1)	1 (?)	–	–
Latridiidae	–	3 (1)	–	–
Limnichidae	–	1 (1)	–	–
Lophocateridae	–	2 (1)	–	–
Meloidae	2 (2)	3 (2)	–	–
Melyridae	1 (1)	10 (2)	(1)	(0.7%)
Monotomidae	–	1 (?)	–	–
Mordellidae	–	5 (3)	–	–
Mycetophagidae	1 (1)	1 (1)	–	–
Nitidulidae	–	3 (2)	–	–
Oedemeridae	2 (2)	5 (4)	(1)	(0.7%)
Phalacridae	1 (1)	3 (3)	–	–
Ptinidae	4 (4)	26 (16)	1 (2)	0.6% (1.4%)
Rhadalidae	–	2 (?)	–	–
Rhipiceridae	–	1 (1)	–	–
Ripiphoridae	–	2 (2)	–	–
Scarabaeidae	13 (11)	37 (16)	2 (5)	1.1% (3.4%)
Scirtidae	–	1 (1)	–	–
Scraptiidae	2 (1)	1 (1)	–	–
Silvanidae	1 (1)	3 (2)	(1)	(0.7%)
Sphindidae	–	1 (1)	–	–
Staphylinidae	9 (5)	73 (36)	(2)	(1.4%)
Tenebrionidae	22 (18)	77 (31)	3 (9)	1.7% (6.2%)
Trogidae	2 (1)	1 (1)	–	–
Zopheridae	1 (1)	3 (3)	–	–
Total	179 (145)	645 (343)	28 (70)	15.6% (48.3%)

(?) unidentified genera in the Socotran fauna. (source of Socotran data based on Hájek and Bezděk (2019)).

In conclusion, the beetle fauna of Farasan Archipelago of the southern Red Sea, KSA, is a subset of the beetles of KSA mainland and dominated by Saharo-Arabian and Afrotropical species. Only 15.6% of its species are shared with the Socotra Archipelago. The new records for the Arabian Peninsula and KSA enrich knowledge of the region’s fauna and also indicate that further studies on the beetles of KSA are still needed.
