# Corrigenda: The first survey of the beetles (Coleoptera) of the Farasan Archipelago of the southern Red Sea, Kingdom of Saudi Arabia. ZooKeys 959: 17–86. https://doi.org/10.3897/zookeys.959.51224

**DOI:** 10.3897/zookeys.1005.61812

**Published:** 2020-12-18

**Authors:** Mahmoud S. Abdel-Dayem, Jiří Háva

**Affiliations:** 1 King Saud University Museum of Arthropods (KSMA), Plant Protection Department, College of Food and Agriculture Sciences, King Saud University, Riyadh, 11451, Saudi Arabia King Saud University Riyadh Saudi Arabia; 2 Forestry and Game Management Research Institute, Strnady 136, CZ-252 02 Praha 5 - Zbraslav, Czech Republic Forestry and Game Management Research Institute Zbraslav Czech Republic

In Abdel-Dayem MS, Abu El-Ghiet UM, Elsheikh TM, Elgharbawy AA, Al-Fifi ZIA, Aldhafer HM (2020) The first survey of the beetles (Coleoptera) of the Farasan Archipelago of the southern Red Sea, Kingdom of Saudi Arabia. ZooKeys 959: 17–86.

After the publication of the work referenced above, our attention was drawn to misidentified species of Dermestidae*Attagenusobtusus* (Gyllenhal in Schönherr, 1808). The correct identification for this species is *Orbeolahirsutulum* (Reiche in Mulsant et Rey, 1868). Other corrections are as follows:

**Page 1, “Abstract” line 13**: “… Arabian and Afrotropical elements (74 spp., 41.0%).” should be

“….. Arabian and Afrotropical elements (75 spp., 41.9%).”

**Page 34, line 25**: “**41. *Attagenusobtusus* (Gyllenhal in Schönherr, 1808)**” should be

“**41. *Orbeolahirsutulum* (Reiche in Mulsant et Rey, 1868)**”

**Page 34, line 28**: The paragraph under “Literature records” should read:

“Riyadh (Mroczkowski 1979).”

**Page 34–35, line 32**: The paragraph under “General distribution” should read:

“AFR-SAR species, distributed in East Africa (Eritrea); Levant, Turkmenistan and Arabian Peninsula (KSA, United Arab Emirates, Yemen).”

**Page 77, line 10**: “that 41.3% of the species belong to Saharo-Arabian …..“ should be

“that 41.9% of the species belong to Saharo-Arabian ….”

**Page 79, lines 4–6**: “… whereas only 28 of Farasan Archipelago’s 179 species (15.6%) are shared with the 645 species currently known from the Socotran fauna, half (50%, 14 spp.) of which ….” should be

“… whereas only 29 of Farasan Archipelago’s 179 species (16.2%) are shared with the 645 species currently known from the Socotran fauna, about half (50%, 15 spp.) of which ….”

**Page 79, line 16**: “Only 15.6% of its species are shared …..” should be

“Only 16.2% of its species are shared …..”

**Page 79, Table 1**: In 20^th^ row 20 “Dermestidae”: 4^th^ column: “1 (3)” should be “2 (3) and 5^th^ column: “0.6% (2.1%)” should be “1.1% (2.1%)”
